# Estimating the population abundance of tissue-infiltrating immune and stromal cell populations using gene expression

**DOI:** 10.1186/s13059-016-1070-5

**Published:** 2016-10-20

**Authors:** Etienne Becht, Nicolas A. Giraldo, Laetitia Lacroix, Bénédicte Buttard, Nabila Elarouci, Florent Petitprez, Janick Selves, Pierre Laurent-Puig, Catherine Sautès-Fridman, Wolf H. Fridman, Aurélien de Reyniès

**Affiliations:** 1 INSERM UMR_S 1138, Cancer, Immune Control and Escape, Cordeliers Research Centre, Paris, France; 2 Université Paris Descartes, Paris, France; 3 Université Pierre et Marie Curie, Paris, France; 4 Programme Cartes d’Identité des Tumeurs, Ligue Nationale Contre le Cancer, Paris, France; 5 Centre de Recherche en Cancérologie de Toulouse, Unité Mixte de Recherche, 1037 INSERM, Université Toulouse III, Toulouse, France; 6 Department of Pathology, Centre Hospitalier Universitaire de Toulouse, Toulouse, France; 7 INSERM, UMR_S1147, Paris, France

**Keywords:** Transcriptomic markers, Gene signatures, Deconvolution, Tumor microenvironment

## Abstract

We introduce the Microenvironment Cell Populations-counter (MCP-counter) method, which allows the robust quantification of the absolute abundance of eight immune and two stromal cell populations in heterogeneous tissues from transcriptomic data. We present in vitro mRNA mixture and ex vivo immunohistochemical data that quantitatively support the validity of our method’s estimates. Additionally, we demonstrate that MCP-counter overcomes several limitations or weaknesses of previously proposed computational approaches. MCP-counter is applied to draw a global picture of immune infiltrates across human healthy tissues and non-hematopoietic human tumors and recapitulates microenvironment-based patient stratifications associated with overall survival in lung adenocarcinoma and colorectal and breast cancer.

**Electronic supplementary material:**

The online version of this article (doi:10.1186/s13059-016-1070-5) contains supplementary material, which is available to authorized users.

## Background

The abundance of tissue-infiltrating immune and non-immune stromal cell populations is highly informative of the types of inflammatory, angiogenic, and desmoplastic reactions occurring in a diseased tissue. In cancer, multiple studies focusing on a limited number of immune populations have reported an association between the extent of infiltrating immune cells and prognosis (reviewed in [1]). For instance, it has been shown that T-cell infiltration correlates with favorable outcome in colorectal [2, 3] and many other cancers [4] and with poor outcome in clear-cell renal cell carcinoma [5]. Other immune cell types, such as macrophages, B cells and natural killer (NK) cells [1], as well as endothelial cells and fibroblasts [6, 7], have been shown to positively or negatively influence the prognosis of cancer patients. Yet, as quantifying just a single cell population is demanding, little is known about how all these cell populations collectively predict the prognosis of cancer patients. The ability to simultaneously quantify multiple cell populations within a tissue sample thus appears critical to identify clinically relevant classes of diseased tissues based on their inflammatory and stromal profiles.

In cellularly heterogeneous tissue samples, transcriptomic measurements average signals originating from the distinct underlying cell populations. The deconvolution of these signals can yield estimates of cell population proportions in a sample [8]. Tens of thousands of transcriptomic profiles are readily available for retrospective analyses from public repositories such as Gene Expression Omnibus (GEO), ArrayExpress, or The Cancer Genome Atlas (TCGA). Different transcriptome-based computational methods were recently proposed to characterize the proportions of immune populations within leukocytes but omit the critical parameter of the overall abundance of leukocytes in the sample [9, 10]. Other published methods lack quantitative validation [11] or are limited to only two cell populations [12]. To overcome these limitations, we introduce Microenvironment Cell Populations (MCP)-counter, a transcriptome-based computational method that robustly quantifies the abundance of immune and non-immune stromal cell populations in a heterogeneous tissue sample.

MCP-counter is available as an R package. From a gene expression matrix, it produces for each sample an abundance score for CD3^+^ T cells, CD8^+^ T cells, cytotoxic lymphocytes, NK cells, B lymphocytes, cells originating from monocytes (monocytic lineage), myeloid dendritic cells, neutrophils, as well as endothelial cells and fibroblasts. MCP-counter estimates are “single sample” scores, in the sense that they are computed on each sample independently. These scores can then be used for direct comparisons of the abundance of the corresponding cell type across samples within a cohort. We show that our approach either complements or outperforms previously published methods. We quantitatively validated MCP-counter both in vitro, by using mRNA mixtures, and ex vivo, by using immunohistochemical cell quantifications on paraffin-embedded tissue sections. We illustrate its application to assess tissue-infiltration in 47 healthy tissue types and in 32 non-hematological malignancies. We show that our method is able to reproduce immunological and stromal prognostic classifications in lung adenocarcinoma and colorectal and breast cancers.

## Results

### Development and validation process of the MCP-counter method

**Fig. 1:**
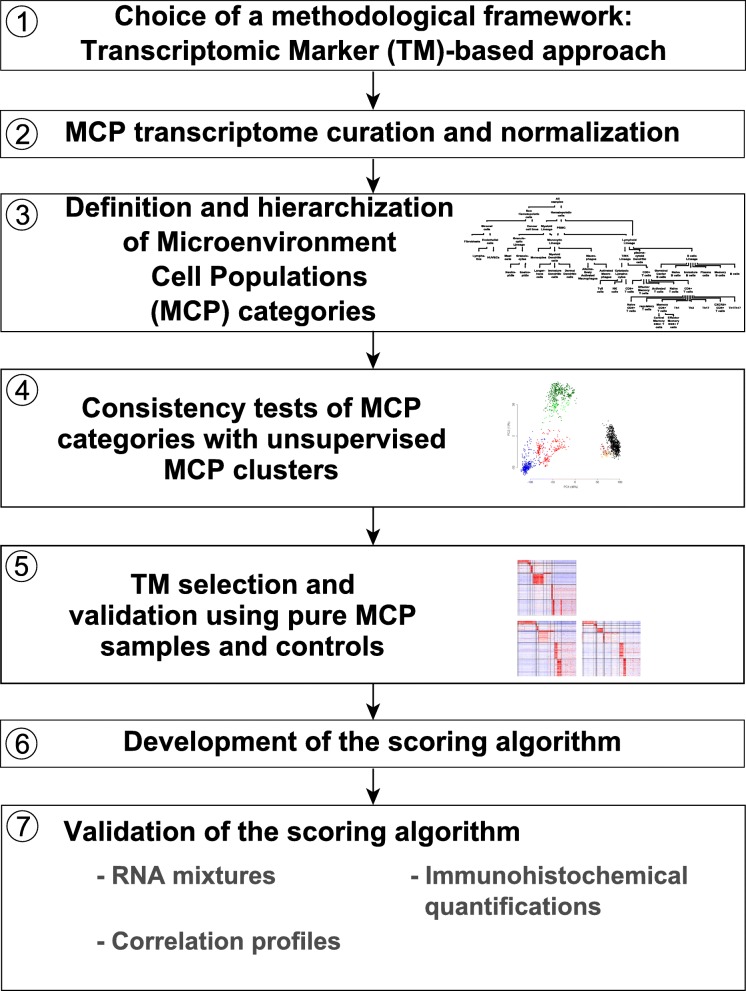
Purpose and development of the MCP-counter method. **a** Comparison of MCP-counter estimates and CIBERSORT-estimated leukocytic fractions. **b** Framework of MCP-counter’s development and validation

**Fig. 2:**
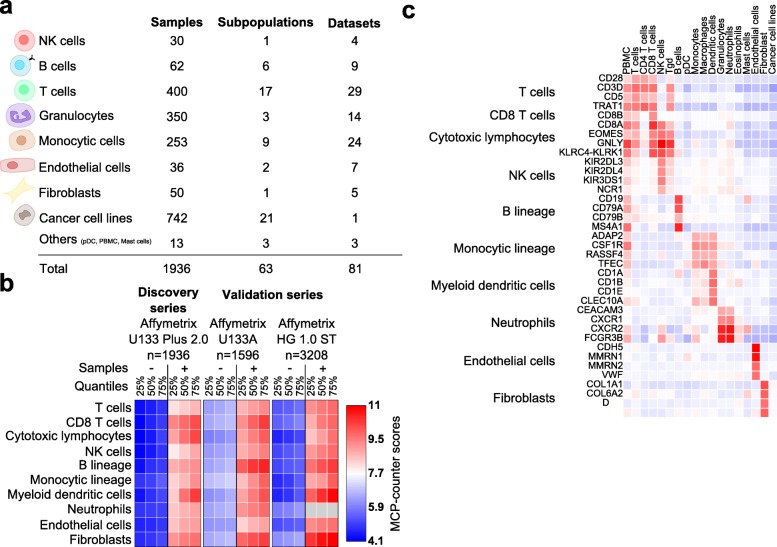
Identification and qualitative validation of transcriptomic markers. **a** The MCP discovery series. *pDC* plasmacytoid dendritic cell, *PBMC* peripheral blood mononuclear cell. **b** Quartiles of MCP-counter scores on positive and control samples in the discovery and validation microenvironment series. *Gray* indicates missing values. **c** Representative transcriptomic markers and their corresponding expression patterns in the MCP discovery series

We designed a method, called MCP-counter, with the objective of measuring the inter-sample relative abundance of different cell populations in a microenvironment. To develop and validate our method, we designed a seven step strategy (Fig. 1). MCP-counter is based on the methodological framework [8] of transcriptomic markers (TM), defined as gene expression features expressed in one and only one cell population and whose expression shows little variation within the population of interest (step 1). It directly follows that, within a sample composed of many different cell populations, the abundance of the population of interest is proportional to the sample’s expression of its related TM. Given their restrictive definition, TM are not guaranteed to exist for all populations. We set up a discovery series by curating Microenvironment Cell Population (MCP) transcriptomes from 81 public datasets derived using Affymetrix Human Genome U133 Plus 2.0 microarrays (step 2). These transcriptomes were normalized [13] to allow their integration into a large meta-dataset which includes 1194 immune or non-immune microenvironment cell population samples; 742 tumor cell line samples were included as negative controls (Additional file 1: Table S1). No hematopoietic tumor cell line was included as a negative control as the transcriptome of these cells is closely related to that of non-malignant hematopoietic cells [14]. After manual curation, all the samples were annotated using 63 labels, of which 42 correspond to microenvironment cell populations and 21 represent cell lines from 21 non-hematopoietic cancer types (Fig. 2a). We organized the 63 cell populations into a pyramidal graph according to rules of inclusion (Additional file 2: Figure S1). We added hematopoiesis-inspired (e.g., lymphoid and myeloid lineages) or functional (e.g., cytotoxic lymphocytes) categories to this pyramid, resulting in a total of 67 nodes corresponding to potential cellular populations in the tissue microenvironment (step 3) (Additional file 2: Figure S1). This organization was validated by its consistency with clusters obtained with transcriptome-based principal component analysis (step 4; Additional file 2: Figure S2).

To identify TM of a given cell population (a node in our cell population pyramid; step 5), we defined as “positive” the samples included in this population and we defined as “negative” the samples that do not contain this population. Samples containing both positive and negative cells are omitted from the analysis for this node. Three criteria were then calculated for each feature (probe set) within the discovery set: a) the mean log_2_-expression difference between positive and negative samples (a threshold of 2 was applied); b) the area under the ROC curve (AUC) of the feature for the identification of the positive samples (threshold of 0.97); and c) a measure of the signal to noise ratio between positive and negative samples (threshold of 1.5) (“Methods”; Additional file 1: Table S2). Gene expression features that reached the defined thresholds simultaneously for all three criteria were retained as TM for the corresponding cell population.

Since we had no a priori knowledge of the populations for which TM could be identified, we applied our selection procedure exhaustively for each non-root node of the sample pyramid (Additional file 2: Figure S1) and selected a posteriori the most relevant TM sets. The number of identified markers at each level of this pyramidal graph is reported in Additional file 1: Table S3. From the 67 nodes, we retained TM for the most precise populations for which TM could be robustly identified. We thus discarded those for which appropriate negative controls were not publically available (for instance, identifying TM for effector memory CD4 T cells at least requires negative controls such as central memory CD4 T cells and effector memory CD8 T cells), those with few positive samples, or those with no identified markers after the selection procedure. Nodes corresponding to more general populations (for instance, lymphocytes or myeloid cells) were discarded as TM for more precise daughter cell populations were available (reasons for discarding each non-selected TM sets are given in Additional file 1: Table S3). We thus retained TMs specific for ten distinct populations: eight immune cell populations (T cells, CD8^+^ T cells, NK cells, cytotoxic lymphocytes, B cell lineage, monocytic lineage cells, myeloid dendritic cells, and neutrophils) and two non-immune stromal populations (endothelial cells and fibroblasts). The 81 datasets from the discovery set spanned 344 different culture conditions, purification methods, and cell treatments, which ensures that the selection of TM was not sensitive to experimental conditions. MCP-counter scores were defined as the log2 average expression of the TM for each population (step 6). We then validated MCP-counter (step 7).

### Qualitative validation of the identified TM

The reproducibility of the identified TM was assessed on two micrenvironment validation series of 1596 samples hybridized on Affymetrix U133A arrays and 3208 samples hybridized on Affymetrix HuGene 1.0ST arrays (Additional file 1: Tables S4 and 5). For the ten cell populations, the specific expression patterns obtained on the discovery series were consistently reproduced (Additional file 2: Figure S3), and the same selection criteria applied to MCP validation series identified significantly overlapping TM sets (Additional file 1: Table S3; *p* < 0.003 for any selected TM set). MCP-counter scores exhibit a clear separation of the cell types in the discovery and two validation series (Fig. 2b), with an AUC above 0.994 for each signature on the 4804 validation samples (Additional file 2: Figure S4).

Although their identification is data-driven, and not knowledge-driven, the selected TM largely overlap with known markers of the corresponding cell populations. They include, for instance, probe sets mapping to CD3D and CD5 for T cells, CD8B for CD8^+^ T cells, EOMES and GNLY for cytotoxic lymphocytes, NCR1 (NKp46) and KIR genes for NK cells, CD19, CD79A, and CD79B for B cells, CSF1R for monocytic cells, CD1 molecules for myeloid dendritic cells, FCGR3B and CEACAM3 (CD66b) for neutrophils, VWF (von Willebrand factor) and CDH5 (VE-cadherin) for endothelial cells, and DCN and TAGLN for fibroblasts (Fig. 2c). In contrast, the screening process excluded from TM some genes usually considered as specific for a given cell population, such as BLK, which we found overexpressed not only in B cells but also at a lower level in plasmacytoid dendritic cells, a result which supports the relevance of a data-driven approach (Additional file 2: Figure S5).

Since, for a given cell population, the TM are expected to be coordinately expressed, we examined the correlation matrices of the TM on the three MCP datasets. We observed highly positive correlation matrices for all populations in the MCP datasets (Additional file 2: Figure S6). Having assessed the reproducibility of this approach, we reduced the number of markers for four populations (neutrophils, fibroblasts, endothelial cells, B cell lineage) which had large TM sets (>90 TM) using information from the MCP validation series (“Methods”). We examined the correlation patterns of these final TM sets in 9408, 3548, and 6451 tumor transcriptomic samples (Additional file 1: Table S6) obtained using Affymetrix Human Genome U133 Plus 2.0, Affymetrix 133A, and Illumina HiSeq transcriptomic platforms, respectively (Additional file 1: Tables S7–S9). We observed largely positive-valued correlation matrices for all MCP in the three tumor datasets, in both pan-cancer (Additional file 2: Figure S7) and single-cancer (Additional file 2: Figure S8) datasets.

### Quantitative validation of MCP-counter abundance estimates

**Fig. 3:**
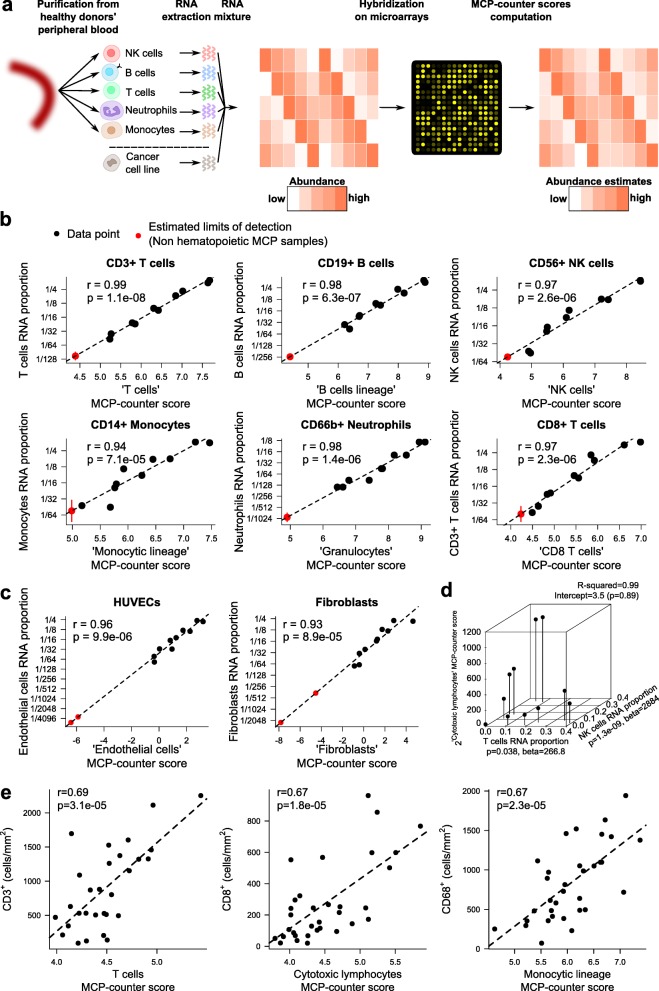
Quantitative validation of MCP-counter estimates. **a** Design of the in vitro RNA mixture validation experiment. **b** Correlation of MCP-counter scores to samples’ known RNA proportions in RNA mixture samples. The *dashed line* represents the least-square regression line. The *red dots* correspond to limits of detection (average score of non-hematopoietic discovery MCP samples on the *x-axis* and corresponding mRNA fraction predicted by this linear regression on the *y-axis*). **c** Endothelial cells and fibroblasts were tested in the same fashion as in **b** but using qPCR data. *HUVEC* human umbilical vein endothelial cell. **d** Three-dimensional scatterplot showing the relationship between the cytotoxic lymphocyte MCP-counter score and T and NK cell proportions in the mixtures. **e** Correlation of MCP-counter scores with corresponding cell densities measured by immunohistochemistry

We quantitatively validated the MCP-counter method. For this purpose, we designed an in vitro RNA mixture experiment (Fig. 3a). Immune populations were purified from healthy donors’ peripheral blood; their RNAs were extracted and mixed in highly variable concentrations (from 0.7 to 46 % of the sample’s RNA). The RNA proportions of the populations were arranged in two transposed latin squares to avoid collinearity and thus ensure specificity (Additional file 1: Table S10). The mixtures were further diluted in a fixed amount of a solution containing mRNA extracted from HCT-116, a colorectal cancer cell line. Transcriptome analyses revealed that MCP-counter scores were highly correlated with the cell proportions for the populations introduced in the mixtures (Fig. 3b), with Pearson’s correlation coefficients ranging from 0.94 to 0.99. We added to these mixtures mRNA extracted from fibroblasts and human umbilical vein endothelial cells, extending the latin square layout (the resulting concentrations are shown in Additional file 1: Table S11), and computed MCP-counter estimates using quantitative polymerase chain reaction (qPCR). For these two cell populations, we also obtained positive and significant linear correlation estimates (Fig. 3c; r = 0.96 and *p* = 9.9 × 10^−6^ for endothelial cells, r = 0.93 and *p* = 8.9.10^−5^ for fibroblasts). Finally, although the amount of cytotoxic lymphocyte mRNA was not controlled in our mixture experiment, we tested whether the CD3+ T cells (through their CD8+ subset) and the NK cells contributed to the level of the cytotoxic lymphocyte MCP-counter score. We thus performed a linear model (“Methods”) which revealed that both cell types contributed significantly and positively to the level of the cytotoxic lymphocyte score, jointly explaining 99 % of the observed variance (Fig. 3d; *p* = 1.3 × 10^−9^ for NK cells and *p* = 0.038 for CD3+ T cells, R^2^ = 0.99). In addition, immunohistochemical (IHC) digital quantification of CD3^+^, CD8α^+^, and CD68^+^ cell densities were performed on tissue sections from 38 colorectal cancer tumors. The IHC-measured density of each cell population was found to correlate with the corresponding MCP-counter score (Fig. 3e).

Finally, we assessed the limit of detection of the technique for each cell population using non-hematopoietic control samples. For each assayed population, we observed a limit of detection below 2 % (depending on the population, from 1/950 to 1/50 of the sample’s total RNA; Fig. 3b). Altogether, these results validate the use of the MCP-counter method to directly compare the abundance of the corresponding cell population across transcriptomic samples.

### Comparison of MCP-counter with previously published methods

**Fig. 4:**
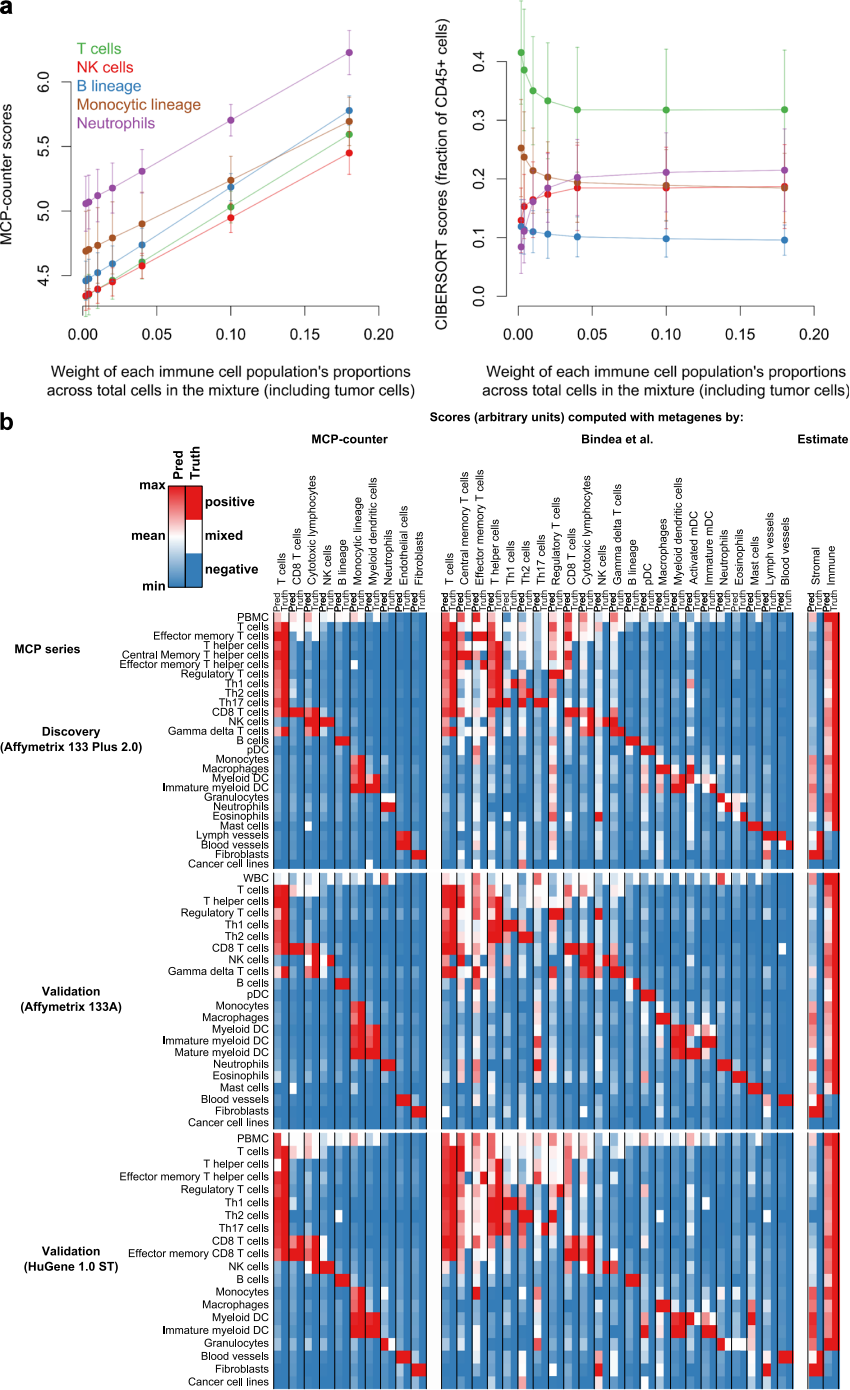
Comparison of MCP-counter with previously published methods. **a** MCP-counter scores (*left*) and CIBERSORT estimates (*right*) on simulated mRNA mixtures where five immune populations are introduced in equal proportions, with a varying proportion of tumor cells (*x-axis*). *Error bars* represent standard error estimates. **b** Heat maps showing scores computed with TM sets from three sources (*Pred columns*). The *Truth columns* indicate the status of the sample for each TM set. Thus, the performance of a TM set is indicated by the concordance between its Pred and Truth columns. The complete sets of markers, which do not use information from validation series, were used for MCP-counter

MCP-counter differs from methods such as CIBERSORT [9], which aims to measure intra-sample (within-leukocyte) proportions of immune cell populations, while MCP-counter outputs an abundance estimate per cell population that enables an inter-sample comparison, at the cost of being expressed in arbitrary units. To illustrate these differences, we simulated mRNA mixtures where the within-leukocyte proportion of five immune cell populations was kept constant and equal while the proportions of tumor cells varied (Fig. 4a). In this setting, only MCP-counter was able to accurately reflect the difference in immune cell abundances across simulated mixtures, while CIBERSORT (accurately) estimated stable proportions of each immune cell population within the leukocytic fraction of the simulated mixtures.

To compare the robustness of the TM sets used in MCP-counter and those identified in previously published methods, we computed metagene scores on the three microenvironment series with TM sets reported by Bindea et al. [11] or Yoshihara et al. [12]. MCP-counter scores achieved high specificity and sensitivity for each of the corresponding cell populations, while some TM sets from other methods were sometimes highly expressed in negative samples (Fig. 4b; Additional file 2: Figure S9).

### Analysis of the microenvironment of normal and cancer tissues

**Fig. 5:**
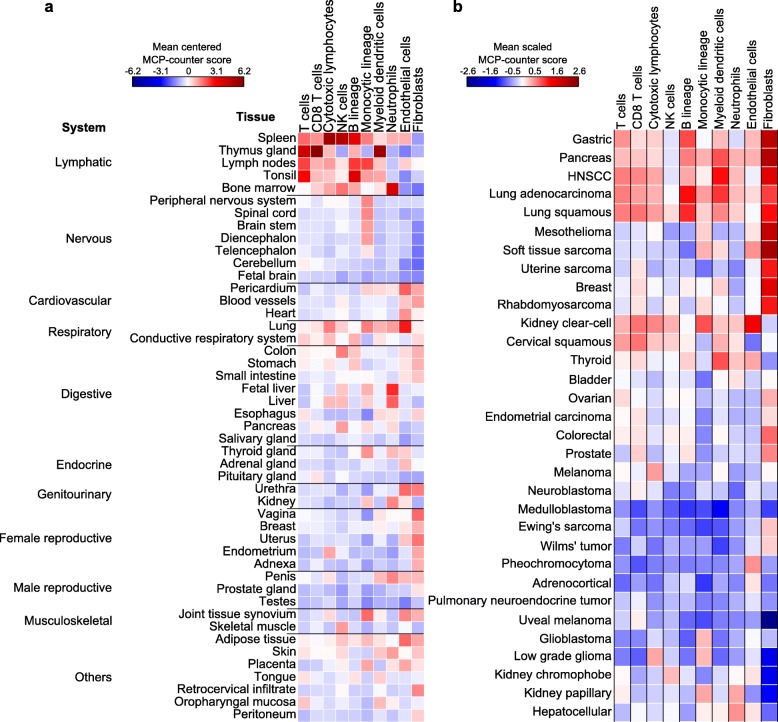
Estimation of the abundance of infiltrating immune and stromal cells across healthy tissues and non-hematopoietic human tumors. **a** MCP-counter scores across healthy tissues. In the case of multiple samples originating from the same type of non-diseased tissue, the resulting MCP-counter scores were averaged. **b** Means of MCP-counter scores across malignant tissues and three transcriptomic platforms. *Rows* are ordered using a hierarchical clustering procedure, with Ward’s aggregation criterion and Euclidean distance. *HNSCC* head and neck squamous cell carcinoma

We applied MCP-counter to a first dataset of 505 samples spanning 47 non-pathological anatomical locations [15]. Lymphoid organs (spleen, lymph nodes, tonsils, bone marrow) were, as expected, found to harbor a high number of immune cells, while the thymus featured the highest MCP-counter score for CD3^+^ T cells (Fig. 5a). On the other hand, known “immune sanctuaries”, such as testes, were correctly found to feature little abundance of immune cells.

Tumor immunology is one of the natural applications of MCP-counter. We estimated the abundance of immune and non-immune cell populations in 19,407 samples spanning 32 non-hematopoietic human tumors, thus proposing a global analysis of non-malignant cell population abundances across human cancers (Fig. 5b). The MCP-counter scores obtained from the three underlying transcriptome platforms (Affymetrix Human Genome U133 Plus 2.0, Affymetrix 133A, and Illumina HiSeq) yielded reproducible patterns across cancer types (Additional file 2: Figure S10) and were thus averaged to produce a synthetic view (Fig. 5b). We observed that kidney clear-cell carcinoma had the highest abundance of endothelial cells of all cancers, while uveal melanomas, which occur in the eye, an immune sanctuary, are poorly infiltrated by immune cells. Colorectal cancer samples were average in terms of immune cell abundance. Gliomas and glioblastomas appeared poorly infiltrated by T cells. The often viral-induced cervical squamous carcinoma is highly infiltrated by cytotoxic T and NK cells but poorly by cells of monocytic origin.

### Prognostic value associated with MCP-counter estimates

**Fig. 6:**
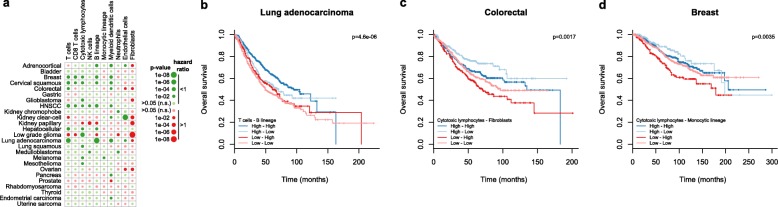
Prognostic value associated with MCP-counter scores in non-hematopoietic human cancers. **a** Meta-analysis for the univariate prognostic value for overall survival of MCP-counter scores in human cancers. *Bigger circles* represent lower *p* values. *Green* represents hazard ratios lower than 1 (favorable prognostic impact) and *red* hazard ratios higher than 1 (poor prognostic impact). *Dull colors* represent *p* values higher than 0.05. *HNSCC* head and neck squamous cell carcinoma. **b**–**d** Tumor microenvironment classifications of **b** lung adenocarcinomas based on the abundance of infiltrating T and B cells, **c** colorectal cancer based on the abundance of cytotoxic lymphocytes and fibroblasts, and **d** breast cancer based on the abundance of cytotoxic lymphocytes and cells of monocytic origin

The three tumor series included, respectively, 2631, 1615, and 6047 samples that had been annotated for overall survival (OS). We additionally curated 1591 tumor transcriptomes with OS annotations obtained using other transcriptomic platforms (Additional file 1: Tables S6 and S12). By performing a meta-analysis of univariate Cox models adjusted on each independent study, we assessed the correlation between the abundance of each microenvironment cell population, as estimated by MCP-counter scores, and OS within cancer types (Fig. 6a). Although this univariate analysis was not adjusted for variables potentially influencing OS, such as tumor stage or treatments received, it nonetheless appeared consistent with the published literature [1], notably revealing an overall favorable prognosis associated with infiltration by T cells, except in kidney clear-cell carcinoma as previously reported [5] and in low grade glioma. Fibroblasts were mostly associated with poor outcome.

To assess whether MCP-counter was relevant to identify tumor subgroups based on their relative infiltration by multiple cell populations, we attempted to reproduce previously reported prognostic classifications. In lung non-small cell adenocarcinoma, it was recently reported that infiltration by B or T cells independently predicts favorable prognosis [16, 17]. In colorectal cancer, extensive literature exists about the protective role of a high infiltration by T cells [2, 3], while fibroblasts were reported to be associated with poor outcome [6, 7]. In breast cancer, a stratification relying on cytotoxic T cells (associated with favorable outcome) and macrophages (poor outcome) was proposed [18]. Using MCP-counter, we were able to reproduce these clinically relevant patterns (Fig. 6b–d).

## Discussion

Tissue-infiltrating immune and non-immune stromal cells contribute to the measured signal in gene expression experiments. Retrieving this information can yield estimates of the abundance of tissue-infiltrating cells [19], illustrated here in cancer samples. To harness this information, we developed the MCP-counter method, implemented in an easy-to-use R package.

It produces a score for each of ten distinct MCP. We validated that these scores are accurate abundance estimates in three different settings: a) transcriptomic profiles of 4804 validation MCP samples, in which the MCP-counter score separated positive and negative samples (relative to each of the ten cell populations) with high specificity and sensitivity; b) in an in vitro RNA mixture setting, where we showed that MCP-counter scores corresponding to the cell populations from which RNAs were extracted highly correlated (Pearson’s correlation coefficients ranging from 0.93 to 0.99) with the RNA fraction of the corresponding cell population in the mixture; and c) in an ex vivo setting where we showed that MCP-counter estimates correlated with IHC measurements of the corresponding cell densities. Using the in vitro setting, we showed that MCP-counter’s lower limit of detection for a population was below 2 % of the sample’s total RNA proportion when using Affymetrix Human Genome U133 Plus 2.0 microarrays. This limit of detection might be lowered by using more sensitive gene expression techniques, Nanostring, or RNA-sequencing assays. We consistently observed lower limits of detection using qPCR data for two cell populations (Fig. 3c).

Other techniques to quantitatively characterize the cellular composition of a heterogeneous tissue notably include flow cytometry and enzymatic IHC. MCP-counter estimates are conceptually close to IHC-estimated cell densities (number of cells per surface unit on a tissue section), as the produced estimates can be used to compare the abundance of the corresponding cell populations across samples. Unlike IHC, however, MCP-counter enables the simultaneous quantification of ten cell populations with a single gene-expression experiment, while IHC quantifications are usually limited to a couple of markers. Information of the cells’ spatial localization, which is available in IHC experiments, is lost, however, when using transcriptomic technologies. Histological confirmation of MCP-counter estimates may thus be necessary in cases where contamination of samples by surrounding tissues is unavoidable. DNA-sequencing data could also be leveraged to estimate the proportion of cells with rearranged T-cell receptor or B-cell receptor loci, providing information about both the abundance and repertoire of these two populations. The eight other populations for which MCP-counter provides estimates are, however, unquantifiable using DNA-sequencing data. Studying the clonality of T and B cells is, however, an interesting covariate to complement abundance estimates of these cell populations and is accessible from RNA-seq data, as recently demonstrated in tumor samples [20–22].

MCP-counter is more sensitive and specific in the interpretation of its scores than other previously published TM-based methods [11, 12] as a result of the rigorous, unbiased, and conservative approach to define the TM sets on which it is based (Fig. 4b) and, importantly, has been quantitatively validated experimentally (Fig. 3). It conceptually differs from flow cytometry experiments or flow cytometry-inspired computational methods such as CIBERSORT [9], which aim at describing the relative proportions of various cell populations within a single sample (Fig. 4a). In contrast, MCP-counter is specifically designed to compare the absolute abundance of a given cell population across multiple samples.

MCP-counter scores linearly correlate with the corresponding cell population abundances across samples, but they are expressed in arbitrary units. These arbitrary units are dependent on the gene expression platform used to produce the data and one can only compare samples produced with the same gene expression platforms. Nonetheless, we showed that the relative cellular abundance across three large tumor datasets, totaling more than 19,000 tumors and obtained with three different gene expression platforms, are largely consistent (Additional file 2: Figure S10), validating the use of MCP-counter to assess which samples are most or least infiltrated by each characterized cell population. Nonetheless, since MCP-counter scores are based on summarized gene expression features (such as reads per kilobase per millions), its accuracy may suffer if the quality of this summary is low. The cell populations whose abundance is estimated by MCP-counter are usually at relatively low frequencies in tissue samples. Thus, sequencing samples at high depth (>80 million reads per sample) [23], which has been reported to improve the quantification of rare transcripts, may improve the accuracy of MCP-counter estimates from RNA-sequencing samples.

We illustrated the use of MCP-counter on non-diseased human tissues and observed abundance estimates consistent with the known immunological status of the samples. We applied MCP-counter to describe the average MCP cellular abundances in 32 non-hematopoietic human malignancies. This analysis confirmed the very high vascularization of clear-cell renal cell carcinoma and showed that cervical squamous cell carcinoma tumors, which are often virally induced, are highly infiltrated by T lymphocytes and, notably, cytotoxic T cells but only moderately by other immune subsets. Other results appeared more surprising, such as the high abundance of fibroblastic cells in the microenvironment of stromal tumors—which may originate from a subset of dedifferentiated tumor cells or the relatively low vascularization of hepatocellular carcinoma samples—which is possibly due to the unique phenotype of endothelial cells in the liver. MCP-counter should in these cases be compared with histopathological knowledge and data within a given cancer.

MCP-counter is most relevant to stratify a cohort of similar samples based on the composition of their immune and stromal microenvironments, or to follow the composition of the microenvironment over time. The use of MCP-counter confirmed that significant univariate associations between OS and tumor infiltration by cytotoxic lymphocytes were mostly positive [4]. In contrast, significant associations between prognosis and extensive abundance of non-immune stromal cell populations and, notably, fibroblasts were shown to be mostly negative using MCP-counter. These observations, largely consistent with the published literature [1, 4, 6, 7], validate the use of MCP-counter to assess the prognostic value of MCP in other cohorts of patients.

MCP-counter complements IHC approaches in that it enables the analysis of ten cell populations using a single gene expression experiment, thus enabling the rapid generation of research hypotheses than can then be confirmed and spatially studied using histological data. We notably illustrated its use to separately classify lung adenocarcinoma, colorectal, and breast tumors into microenvironment-defined subgroups. In this setting, we were able to confirm the prognostic impact of three previously published microenvironment-based tumor classifications. These results suggest that MCP-counter may enable the identification of new multi-marker microenvironmental stratifications.

MCP-counter relies on TM which have been identified in a dataset containing gene expression profiles of cancer cell lines from 21 different anatomic locations among its negative controls, ensuring applicability in a wide range of samples. This large diversity of control samples may, however, discard TM which would be relevant in a specific setting: for instance, the screening procedure discarded NCAM1 (CD56), a widely used marker of NK cells, as it is also expressed by nervous malignant cells and is thus unsuitable to quantify NK cells in brain samples. The general framework that we have developed could thus be tailored to identify additional TM for investigation in a more restricted set of organs.

MCP-counter can potentially be incorporated in clinical routines to characterize immune infiltration in samples where IHC-based quantifications are impossible, such as for fine-needle aspiration biopsies. In this setting, samples are typically collected one at a time. To complement the current multi-sample use of MCP-counter, designed for exploratory analyses, one could notably settle on a desired gene expression platform and use a set of calibrating samples. For instance, the in vitro RNA mixtures described here could help to map MCP-counter abundance scores to non-arbitrary units, such as the percentage of the corresponding cells within a sample. Setting-specific tuning may, however, be required to reach the reliability necessary for clinical protocols.

## Conclusions

To our knowledge, MCP-counter is the first validated computational method that enables the robust quantification of the abundance of multiple immune and non-immune stromal populations in the transcriptome of cellularly heterogeneous tissues such as normal or malignant tissues. It might be relevant in a clinical setting, as immune biomarkers can help to predict a patient’s prognosis [3] or response to therapies [24], most notably to immunotherapies [25]. Its retrospective and prospective application to study inflammatory profiles through transcriptomics should help to unravel the role of immune and stromal populations in cancers and other diseases and to decipher the interplay between these populations.

## Methods

### Gene expression profile datasets

We curated transcriptomic profiles from several types of samples from public repositories (microenvironment cell populations (MCP), non-hematopoietic human tumors, non-diseased human tissues, in vitro RNA mixtures) and obtained using different gene expression platforms (mainly Affymetrix HGU 133 Plus 2.0, HGU 133A, HuGene 1.0 ST, and Illumina HiSeq 2000). For survival analysis, transcriptomic profiles of non-hematopoietic human tumors with OS annotations were also included. Table 1 lists the types of samples curated, stratified by gene expression platforms, and points to the identifiers of the included samples.

**Table 1:** Types of samples curated

Sample type	Gene expression platform	Sample identifiers
Microenvironment cell populations	Affymetrix Human Genome 133 Plus 2.0	Additional file 1: Table S1 GEO accession number GSE86362
Microenvironment cell populations	Affymetrix Human Genome 133A	Additional file 1: Table S4 GEO accession number GSE86363
Microenvironment cell populations	Affymetrix HuGene 1.0 ST	Additional file 1: Table S5 GEO accession number GSE86357
In vitro RNA mixtures	Affymetrix Human Genome 133 Plus 2.0	Additional file 1: Table S11 GEO accession number GSE64385
Non-diseased human tissues	Affymetrix Human Genome 133 Plus 2.0	Additional file 1: Table S14 GEO accession number GSE7307
Non-hematopoietic tumors	Affymetrix Human Genome 133 Plus 2.0	Additional file 1: Table S7
Non-hematopoietic tumors	Affymetrix Human Genome 133A	Additional file 1: Table S8
Non-hematopoietic tumors	Illumina HiSeq 2000	Additional file 1: Table S9
Non-hematopoietic tumors	Other gene expression platforms	Additional file 1: Table S11

### Gene expression profile normalization

#### Affymetrix Human Genome U133 plus 2.0, Human Genome 133A, and HuGene 1.0 ST arrays

MCP datasets and tumor datasets from Affymetrix Human Genome U133 Plus 2.0, Human Genome 133A, and HuGene 1.0 ST arrays were normalized using the frozen robust multiarray average (fRMA) method, implemented in the fRMA R package (version 1.18.0). Unlike RMA, fRMA uses fixed estimates of probe-specific effects and variances, allowing a consistent normalization of gene expression profiles (GEP) from different series, provided that they were obtained on the same gene expression platform.

GEP obtained with the Affymetrix Human Genome U133 Plus 2.0, Human Genome 133A, and HuGene 1.0 ST array platforms were thus normalized using the frma function of the frma Bioconductor R package using the preprocessing input vectors provided by the Bioconductor R packages frmahgu133plus2frmavecs version 1.3.0, frma133afrmavecs version 1.3.0, and hugene.1.0.st.v1frmavecs version 1.0.0, respectively. The frma method was called on batches of CEL files corresponding to individual series.

#### TCGA gene expression data

Gene expression data from non-currently embargoed TCGA projects were obtained from TCGA data portal (https://gdc-portal.nci.nih.gov/). Only GEP obtained using Illumina HiSeq 2000 were retrieved. Already-normalized “level 3” data were downloaded separately for each project. The resulting *.rsem.genes.normalized_results files were then merged into a single pan-cancer expression matrix.

#### Other gene expression platforms

For GEP obtained using other gene expression platforms, we used pre-processed GEP data as published by each study and available from the corresponding public repositories.

### Publicly available sample annotations

#### MCP datasets: discovery series—Affymetrix U133 plus 2.0 arrays)

Samples were annotated according to the author-reported phenotypes, yielding 344 distinct labels (Additional file 1: Table S1). We recoded these labels into 63 categories, including 21 cancer cell phenotypes (42 immune and stromal labels).

#### MCP datasets: validation series—Affymetrix 133A and HuGene 1.0 ST arrays)

Samples were annotated according to the 63 labels used to annotate the discovery microenvironment series. The previously defined 63 categories did not fit the phenotype of a few samples and motivated the addition of 15 categories (Additional file 1: Tables S4 and S5). These newly defined categories all refine some of the initial 63 labels.

#### Non-diseased human tissues (GEO:GSE7307)

Sample annotations were retrieved from GEO:GSE7307. Samples corresponding to diseased tissues, cell lines, or sorted immune cells were discarded, retaining only the non-diseased, cellularly heterogeneous samples. For clarity, some tissues were regrouped in broader anatomical locations (detailed in Additional file 1: Table S14). Anatomical systems were manually added.

#### Non-hematopoietic tumor sample series

From the three tumor datasets, only samples corresponding to primary tumors obtained from tumor resections with no neoadjuvant treatments and that did not use laser capture microdissection were retained for analysis (Additional file 1: Tables S6, S7, S8, S9, and S12).

Annotations for samples retrieved from GEO and ArrayExpress were retrieved from the “Series matrix” files and “sdrf” files, respectively. Annotations for TCGA samples were retrieved from TCGA data portal (https://gdc-portal.nci.nih.gov/).

Since annotations originated from many different groups, only a subset of the variables were retained for each series. The corresponding values were harmonized into a consistent ontology. The following list contains the final set of variables for which sample annotations were retained: sample identifiers; series identifiers; gene expression platform; cancer type; sample type (autopsy, biopsy, cell culture, surgery followed by laser capture microdissection, surgery); overall survival event and delay (months)

#### Non-hematopoietic tumor samples series (other platforms)

Only sample identifiers, tumor types, sample status and OS were retained.

#### Removal of tumor sample duplicates

Some tumor GEP are present in multiple public datasets and correctly labeled as “Reanalyzed”. In this case, only the original sample was considered for analysis. To avoid unspecified duplicated GEP, we computed MD5 checksums for all uncompressed CEL files. Samples with identical MD5 checksums were considered duplicates. In this case, only the sample belonging to the oldest series was kept. Annotations present on the most recent sample instance and absent from the older sample instance were added to the older sample’s annotation. Conflicting annotations were resolved by keeping the oldest annotation. These conflicts never occurred for clinical follow-up variables.

### Selection of the TM

#### Organization of the samples into a pyramidal graph of categories

We define as a pyramid a directed acyclic graph with a root node. Samples of microenvironment purified cells were labeled according to their reported immune or stromal populations, resulting in 63 distinct labels in the MCP discovery series, with an additional 15 labels for the MCP validation series, resulting in a total of 78 labels. We organized these labels in a pyramidal graph (Additional file 2: Figure S1) with nodes representing populations (categories) and directed edges representing relations of inclusion. For instance, the labels “CD8^+^ T cells”, “CD4^+^ T cells”, “Tγδ cells”, “Memory T cells”, “Activated T cells”, and “Naïve T cells” and all labels included in them (for instance “Effector-memory CD8 T cells”) form the “T cells” category, which itself is included in the “T/NK lineage” category. Of these 78 sample labels, some correspond to terminal leaves of this pyramid (e.g., “Canonical CD4 Treg cells”), while others correspond to higher level nodes (e.g., peripheral-blood mononuclear cells (“PBMC”)). In addition to these 78 labels, 15 hematopoiesis or immunology-inspired categories that are not directly represented by samples but relevant for their organization in a structured pyramid (for instance “Lymphocytes”) or as a potential cell population (for instance “antigen-experienced B cells”) were added (Additional file 1: Table S13). Categories corresponding to tumor samples were discarded for the identification of TM and only kept as negative controls, resulting in 68 categories available for screening.

Having defined this set of 78 labels and 68 categories (53 categories are directly represented by labels, with 15 additional categories not directly represented in the dataset), we exhaustively encoded the relationships between labels and categories using three possible relationships (Additional file 1: Table S13). Relative to a category, we define three sets of samples:

- *C* : “positive samples” are those whose label is included in the category (all cells composing a sample which is in *C* are in the category)
- $\overline{C}$ : “negative samples” are those whose label is strictly non-overlapping with the category (all cells of a sample which is in $\overline{C}$ are not in the category)
-1 : “mixed samples” are those whose label is partly overlapping with the category (some cells of the sample are in *C* and some are in $\overline{C}$).

For instance, for CD8^+^ T cells, *C* is the set of samples whose label is “CD8 T cells” or “Effector memory CD8 T cells” (Additional file 2: Figure S1; Additional file 1: Table S13), mixed samples are, for instance, CD3^+^ T cells as they mix CD4^+^ and CD8^+^ T cells, or PBMC as they mix CD8^+^ T cells with, e.g., monocytes. $\overline{C}$ is defined as all non-positive non-mixed samples.

Note that the relationships represented in Additional file 2: Figure S1 only correspond to the “direct inclusion” relationship, which is transitive (we thus removed for clarity all the arrows which can be inferred by transitivity). Hence, strict exclusion or mixture relationships are not represented but are taken into account during the screening process (the related information is available in Additional file 1: Table S13).

#### Selection of TMs for each category

We performed an exhaustive exploration of the category pyramid to screen for the existence of potential TM at each node. For a given node of the pyramid, Additional file 1: Table S13 defines the set of positive samples *C* and negative samples $\overline{C}$ (mixed samples are discarded for this node). Common differential expression tests (based on cutoff values on the fold-change and the significance of, e.g., a Student’s t-test) aim at investigating whether two samples drawn from two continuous distributions have the same mean. Our definition of a TM required a more stringent approach, as we not only required differential expression but also null expression in the negative samples. Given these two sets of samples, a triplet of probe set-level statistics was thus computed per probe set: the positive AUC, the fold change (FC), and a specific fold change (sFC), with the latter two having the following definitions:

$$ FC = X - \overline{X} $$

$$ sFC=\left(X-\overline{X} min\right)/\left(\overline{X} max-\overline{X} min\right) $$

where we denote by X the centroid (i.e., average across all samples) of category *C*, $\overline{\mathrm{X}}$ the centroid of $\overline{C}$, ${\overline{\mathrm{X}}}_{\mathrm{j}}$ the centroid of any class j composing $\overline{C}$ (j = 1..k), $\overline{\mathrm{X}}$ min the minimum value across centroids of classes composing $\overline{C}$ (${\overline{\mathrm{X}}}_{\min }$ = min_j∈1..k_ {${\overline{\mathrm{X}}}_{\mathrm{j}}$}), and $\overline{\mathrm{X}}$ max the max value across centroids of classes composing $\overline{C}$ (${\overline{\mathrm{X}}}_{\max }$= max_j∈1..k_ {${\overline{\mathrm{X}}}_{\mathrm{j}}$}). The specific FC accounts for both a high expression in *C* compared to $\overline{C}$ and a low variability within $\overline{C}$.

For each non-root node of the pyramid, probe sets with AUC >0.97, FC >2, and sFC >1.5 were retained (Additional file 1: Table S3). The choice of the (log_2_) FC cutoff was arbitrarily set to 2, which falls in the upper range of commonly chosen cutoffs for log_2_ FC [26]. We then performed simulations by drawing 2000 “negative” datapoints from N(0,s) and 100 “positive” datapoints from N(2,s), where N(μ,s) designates the normal distribution with mean μ and s is the median standard deviation of probe sets observed in our discovery series, which revealed that an AUC cutoff of 0.97 corresponded to a type two error of 25 %. The cutoff value for the sFC was chosen by examining the univariate distribution of sFC values after filtering probe sets for FC >2 and AUC >0.97 and choosing a cutoff retaining only the upper flat tail of the distribution, with 30 % of the probe sets passing the first two criteria falling above this cutoff.

#### Selection of TM sets for ten populations

Having selected in an unbiased manner the TM for each level of the pyramid, we manually selected the most relevant TM sets, discarding very broad categories (such as “stromal cells” which would, in a tumor, designate all non-malignant cells from the microenvironment), categories with too few (<30) positive samples to reliably identify robust markers from high-dimensional data, those for which no appropriate controls were represented in the discovery series, and those for which no markers were identified.

#### Reduction of TM sets for four populations

For four populations (neutrophils, endothelial cells, fibroblasts, and B lineage cells), the number of TM identified in the discovery series was much higher (>90) than for the other cell populations (<25). To obtain more balanced marker sets, we performed the same selection process on the validation series, obtaining TM sets overlapping with those identified in the discovery series (Additional file 1: Table S3). To reduce the number of markers, we thus took the intersection of the markers across the three microenvironment series (B lineage, fibroblasts), or the discovery and the HuGene 1.0ST series (endothelial cells), or the discovery and the Affymetrix HGU 133A series (neutrophils). Of note, this filtering step was not performed for the figures where data from the MCP validation series were used (Fig. 2; Additional file 2: Figures S3, S4, S6).

### Computation of MCP-counter scores

Given a set of transcriptomic markers of a given category, we computed a corresponding per-sample score, called hereafter a *MCP-counter score*, using the log_2_ geometric mean of this set of markers.

### Principal component analyses of microenvironment samples

For each MCP series, we retained samples belonging to well-characterized hematopoietic lineages (T or NK cells, B cells, monocytic lineage, granulocytes, endothelial cells, fibroblasts) and cancer cell lines. Probe sets were filtered to retain only the those above the 95th variance percentile on each series. Principal component analysis was then performed for each series and data for the first principal two components (those explaining the most variance) are displayed (Additional file 2: Figure S2; cell lineages are color coded).

### Correlation profiles of TM in micrenvironment and tumor datasets

For a given set of TMs, corresponding features were subsetted from the expression matrices of the three microenvironment series and three primary tumor series (Affymetrix Human Genome U133 Plus 2.0, 133A, and TCGA). On these six matrices, Pearson correlation coefficients were computed for each pair of features (Additional file 2: Figures S5–S7).

This analysis is motivated by the following model: our definition of a transcriptomic marker is a feature that is expressed in one and only one cell population at an homogeneous level. Let (i) *S* be a tissue sample composed of *n* cell populations with proportions π_**i**_ (i = 1..n), (ii) *f* be a feature and (iii) *f*_*S*_ be its measured expression in sample S and (iv) *f*_*i*_ its measured expression in a cell population i (i = 1..n). Assuming linearity between the measured expression of the feature f and the corresponding targeted mRNA, we have the following equation:

1 $$ {\mathrm{f}}_{\mathrm{S}}={{\displaystyle {\sum}_{i=1}^n\mathrm{f}}}_{\mathrm{i}}.{\uppi}_{\mathrm{i}} $$

This equation states that the measured signal in the cellularly heterogeneous tissue is sum of its proportion-weighted expression level in each individual population. In the case of a TM, since *f*_*i*_ is non-null in a single population *k*, we have:

2 $$ {f}_S={f}_k.{\pi}_k $$

i.e., the measured expression level is proportional to the proportion of the cell population with non-null expression for the TM.

If *g* is another expression feature that is a transcriptomic marker for population *k*, we also have:

3 $$ {g}_S={g}_k.{\pi}_k $$

Combining Eqs. 2 and 3, we have:

4 $$ {\uppi}_{\mathrm{k}}=\frac{{\mathrm{f}}_{\mathrm{S}}}{{\mathrm{f}}_k} = \frac{{\mathrm{g}}_S}{{\mathrm{g}}_k} $$

If we now have *m* transcriptomic measurements, Eq. 4 stands independently in each measured tissue. We thus have, for each *j* in 1,…,m:

5 $$ {\uppi}_{\mathrm{k},\mathrm{j}}=\frac{{\mathrm{f}}_{\mathrm{S},\mathrm{j}}}{{\mathrm{f}}_k}=\frac{{\mathrm{g}}_{S,j}}{{\mathrm{g}}_k} $$

where ${\pi}_{k,j}$ is the proportion of the cell population *k* in sample *j*, *f*_*S,j*_ is the expression level of feature *f* in sample *j*, and *g*_*S,j*_ is the expression level of feature *g* in sample *j*. Importantly, since *f*_*k*_ and *g*_*k*_ are the expression levels of *f* and *g* in the cell population *k*, they are independent of sample *j*.

Equation 5 thus shows that, for each sample, *f*_*S*_ equals *g*_*S*_ times the constant *f*_*k*_ */g*_*k*_. We thus show that given that *f* and *g* are two transcriptomic markers for population *k*, then the expression of *f* and *g* in a series of transcriptomic measurements is proportional. In a perfect system (in which no non-linear and noise are present), we thus expect correlation coefficients of 1 for two TM specific for a given cell population.

### RNA mixture models

#### Peripheral blood immune cell sorting

Peripheral venous blood was extracted for three healthy donors using heparin vacuntainer tubes (BD Bioscience). Peripheral blood mononuclear cells (PBMC) or polymorphonuclear cells (PMN) were isolated using Ficoll-Paque PLUS (GE Healthcare Life Sience) or Polymorph Prep (Axis-Shield) density gradients, respectively. PBMCs were stained with anti-CD3 FITC (clone UCHT1), anti-CD14 APC (MΦP9), anti-CD19 ECD (J3-119) and anti-CD56 PE (B159); PMNs were stained with anti-CD66b FITC (G10F5), anti-CD19 ECD (J3-119), anti-CD3 PE (UCHT1), anti-CD56 PE (B159), and anti-CD14 APC (MΦP9). Cell sorting was done in a FACS Aria cytometer (BD Bioscience) and cell purity higher than 97 % was always achieved. We sorted the following populations: T cells (DAPI^−^/CD3^+^/CD14^−^/CD19^−^/CD56^−^), monocytes (DAPI^−^/CD3^−^/CD14^+^/CD19^−^/CD56^−^), B cells (DAPI^−^/CD3^−^/CD14^−^/CD19^+^/CD56^−^) and NK cells (DAPI^−^/CD3^−^/CD14^−^/CD19^−^/CD56^+^) on PBMCs, and neutrophils (DAPI^−^/CD66b^+^/CD19^−^/CD3^−^/CD56^−^/CD14^−^) on PMNs.

#### Cell culture

HCT116 and CCD-18Co cells lines were purchased from ATCC and cultured according to the vendor’s instructions. Primary human umbilical vein endothelial cells (HUVEC; third-passage) were isolated as previously described [27] and cultured in M199 medium (Gibco, Paisley, UK) with 20 % fetal calf serum.

#### RNA extraction

Cells were lysed in RLT (QIAGEN)-1 % mercaptoethanol buffer and RNA was purified with a Maxwell 16 simplyRNA Kit (Promega) according to the manufacturer’s instructions. Genetic material quality and quantity were determined with a 2100 Bioanalyzer Instrument (Agilent Technologies).

#### Mixtures of RNA solutions

A set of four twofold serial dilutions were performed on each aliquot of RNA extracted from sorted peripheral blood immune cells, yielding solutions of decreasing concentration S0 to S4 for each cell population. Ten aliquots were used to mix these solutions using two transposed latin square layouts (Table 2).

**Table 2:** mRNA mixtures experimental layout

	Mix 1	Mix 2	Mix 3	Mix 4	Mix 5	Mix 6	Mix 7	Mix 8	Mix 9	Mix 10	Mix 11	Mix 12
NK cells	-	-	S0	S1	S2	S3	S4	S0	S4	S3	S2	S1
B cells	-	-	S4	S0	S1	S2	S3	S4	S3	S2	S1	S0
T cells	-	-	S3	S4	S0	S1	S2	S3	S2	S1	S0	S4
Neutrophils	-	-	S2	S3	S4	S0	S1	S2	S1	S0	S4	S3
Monocytes	-	-	S1	S2	S3	S4	S0	S1	S0	S4	S3	S2
HCT116	10 ng	10 ng	10 ng	10 ng	10 ng	10 ng	10 ng	10 ng	10 ng	10 ng	10 ng	10 ng

The volume corresponding to 10 ng of a solution of HCT116 colorectal cancer cell line-extracted mRNA was then added to mixes 3 to 12. Two additional samples (mixes 1 and 2) of pure HCT116 mRNA were also included. The resulting concentrations are available in Additional file 1: Table S10.

For the qPCR experiments, we added mRNA from fibroblasts and HUVEC to these previously generated mixtures in the following way.

mRNA solutions from mixes 1 to 12 described above where unthawed, to which we added a set of four twofold serial dilutions of mRNa from cultured fibroblasts and HUVEC, yielding solutions of decreasing concentration S0 to S4 for each cell population (Table 3). For these two cell populations, S0 corresponds to 3 ng/μL of mRNA. The resulting proportions are available in Additional file 1: Table S10.

**Table 3:** mRNA mixtures extended experimental layout

	Mix 1	Mix 2	Mix 3	Mix 4	Mix 5	Mix 6	Mix 7	Mix 8	Mix 9	Mix 10	Mix 11	Mix 12
HUVECs	-	-	S4	S3	S2	S1	S0	S0	S4	S3	S2	S1
Fibroblasts	-	-	S3	S2	S1	S0	S4	S4	S0	S1	S2	S3

#### Microarray hybridization

Biotinylated double-stranded cDNA targets were prepared from 10 ng of total RNA using the NuGEN Ovation Pico WTA System V2 kit (catalog number 3302) followed by the NuGEN Encore Biotin Module Kit (catalog number 4200) according to the manufacturer’s recommendations. Following fragmentation and labeling, 4.55 μg of cDNA was hybridized for 16 h at 45 °C, 60 rpm on Human GeneChip Human Genome U133 plus 2.0 arrays (Affymetrix). The chips were washed and stained in a GeneChip Fluidics Station 450 (Affymetrix) using the FS450_0004 script and scanned with a GeneChip Scanner 3000 7G (Affymetrix) at a resolution of 1.56 μm. Raw data (.CEL intensity files) were extracted from the scanned images using the Affymetrix GeneChip Command Console (AGCC) version 4.0.

#### Quantitative PCR

Reverse transcription PCR was conducted with the High-Capacity cDNA Reverse Transcription kit (Applied Biosystem). Quantitative expression analysis of 12 genes was determined on an Applied Biosystems 7900HT Fast Real-Time PCR System. Expression levels of genes were determined using threshold cycle (Ct) values normalized to beta-actin (ΔCt). The list of analyzed genes is displayed in Additional file 1: Table S15.

#### Correlation between MCP-counter scores and known mRNA proportions

MCP-counter scores were computed from the fRMA-normalized RNA-mixture microarray dataset and plotted against the known mRNA log-proportions. Pearson correlation coefficients and the corresponding tests against the t distribution were performed.

For qPCR experiments, MCP-counter was ran using gene symbols as identifiers and the intersection of the “complete” TM sets for fibroblasts and endothelial cells and the gene assayed by qPCR as features.

#### Estimation of the limit of detection

For each of the five sorted cell populations (NK cells, B cells, T cells, monocytes, neutrophils), we fitted a least-square linear regression model of the known mRNA log-proportions with the corresponding MCP-counter score as predictive variable using the ten mRNA mixture samples.

The MCP-counter scores for non-hematopoietic samples from the MCP discovery series were computed. The above-described linear fits were used to predict the mRNA fraction of the five sorted cell populations from the corresponding MCP-counter scores. The exponential of the mean of the estimated log-proportions across non-hematopoietic samples is reported as the estimate of the limit of detection for each immune cell population assayed.

#### Data deposition

The transcriptome data of the 12 mixture samples has been deposited in NCBI’s Gene Expression Omnibus and are accessible through GEO series accession number GSE64385.

#### Linear model correlating the cytotoxic lymphocyte MCP-counter score to NK and T cell abundances

NK and CD3+ T cells are the two populations introduced in our mRNA mixture experiment that contain cytotoxic lymphocytes (as a whole for NK cells and through their CD8+ subset for CD3+ T cells). The model is thus set up to assess whether the observed cytotoxic lymphocyte scores can be explained by the proportions of NK and T cells in our mixture samples.

Let *C*, *NK*, and *T* designate the mRNA amounts corresponding to cytotoxic lymphocytes, NK cells, and CD3+ T cells, respectively. Let *C.MCP* designate the cytotoxic lymphocyte MCP-counter score. We have the following linear relationship at the mRNA level:

$$ C = a{'}_{NK}.NK+a{'}_T.T+b', $$

where *a’*_*NK*_, *a’*_*T*_ and *b’* are scalar constants.

*C* is not controlled for in our dataset. However, under the hypothesis that *C* correlates with the cytotoxic lymphocyte MCP-counter score, and since the mRNA data are in a log_2_ scale, we have:

$$ C = a{.2}^{C.MCP} + b" $$

Combining these two equations, we have:

$$ {2}^{C.MCP} = {a}_{NK}.NK + {a}_T.T + b $$

with *b* = (*b” − b’*)/*a*, *a*_*NK*_ = *a’*_*NK*_/*a*, and *a*_*T*_ = *a’*_*T*_/*a*, which is also a linear relationship. We thus tested it using a bivariate linear model on our dataset.

### IHC-based cellular density estimates

Serial 5-μm formalin-fixed paraffin-embedded tissue sections from colorectal cancer were stained using autostainerPlus Link 48 (Dako). Antigen retrieval and deparaffinization were carried out on a PT-Link (Dako) using the EnVision FLEX Target Retrieval Solutions (Dako). The antibodies used are listed in Additional file 1: Table S16. Peroxidase activity was detected using diaminobenzidine substrate (Dako). Slides stained with anti-CD3, anti-CD8A, and anti-CD68 were digitalized with a NanoZoomer scanner (Hamamatsu). The densities of positive cells in the tumor core were measured using Calopix software (Tribvn, France).

### Mapping of Affymetrix probe sets to gene identifiers and across series

For Affymetrix Human Genome U133 Plus 2.0, 133A, and HuGene 1.0 ST arrays, probe sets were mapped to gene identifiers using annotations provided by Affymetrix version 35.

TCGA GEP are annotated with ENTREZ identifiers which were converted to HUGO symbols using Homo_sapiens.gene_info (ftp://ftp.ncbi.nih.gov/gene/DATA/GENE_INFO/Mammalia/Homo_sapiens.gene_info.gz; retrieved on 20 May 2015).

When assessing the reproducibility of the selection procedure across microenvironment series, mapping between features was performed as follows. Probesets that are shared between the Affymetrix Human Genome U133 Plus 2.0 and 133A platforms were mapped, while the others were ignored. As HuGene 1.0 ST and Human Genome U133 Plus 2.0 Affymetrix platforms do not share any probe set, probe sets of both platforms mapping to the same gene symbols were mapped between the two platforms.

### R implementation of the MCP-counter method

We implemented MCP-counter as an R package called “MCPcounter”. Users should call the “MCPcounter.estimate” function, which takes a normalized gene expression matrix as its first argument and the type of features that should be mapped to selected TM (probe sets, HUGO symbols, ENTREZ ID) as its second argument.

We used mapped TM to “probe sets” to compute MCP-counter scores for Affymetrix Human Genome U133 Plus 2.0 and Affymetrix 133A samples, and HUGO symbol identifiers for samples obtained on Affymetrix HuGene 1.0 ST, Illumina Hiseq, and other gene expression platforms.

This package is available Zenodo (https://doi.org/10.5281/zenodo.61372).

### Simulation of mRNA mixtures (Fig. 4a)

Let’s call *d* a linear scale (genes × samples) GEP matrix, with samples related to six possible phenotypes, five corresponding to immune cell populations (“T cells”, “NK cells”, “B cells”, “monocytic lineage”, “neutrophils”) and one to non-hematopoietic tumor cell lines. Simulations are performed according to the following three-step procedure.

#### Step 1

For each of these six possible phenotypes, randomly choose one GEP in the microenvironment series 3 among corresponding samples and let’s call *d’* the matrix *d* restricted to these six samples (columns).

#### Step 2

From the six randomly selected samples S_1_..S_6_ (one per phenotype) create two sets of virtual GEP, *S* * and *S* ***’*** **,** defined as **S** * = *d* ’. **W** + *ε and* **S** ’ = *log*_2_(*d* ’. **W** + *ε*). ε corresponds to random noise drawn from the standard normal distribution for each gene expression feature of the simulated mixture. *W* is a 6 × 7 (samples × mixing proportions) matrix (Table 4).

**Table 4:** Cell populations’ weights in the simulated mixture

	Weight in mixture 1	Weight in mixture 2	Weight in mixture 3	Weight in mixture 4	Weight in mixture 5	Weight in mixture 6	Weight in mixture 7
S1	0.002	0.004	0.01	0.02	0.04	0.1	0.18
S2	0.002	0.004	0.01	0.02	0.04	0.1	0.18
S3	0.002	0.004	0.01	0.02	0.04	0.1	0.18
S4	0.002	0.004	0.01	0.02	0.04	0.1	0.18
S5	0.002	0.004	0.01	0.02	0.04	0.1	0.18
S6	0.99	0.98	0.95	0.9	0.8	0.5	0.1

In other words, the six randomly selected GEP are linearly convoluted with weights *w*_*i*_ for each of the five immune populations and the complementary weight 1 − 5*w*_*i*_ for the tumor cell population. For each method, seven different simulated mixtures are thus computed per set of randomly chosen samples.

#### Step 3: run CIBERSORT on S* and MCP-counter on S’

Fifty simulations were performed (leading to 350 estimates as seven values of *w* per simulation run were used). Figure 4a represents, for each cell population, the mean MCP-counter scores or CIBERSORT estimates for a given value of *w* across the 50 simulations. For CIBERSORT, non-zero estimates of non-introduced cell populations (for instance, mast cells) were discarded and the remaining estimates were re-normalized to sum to 1. Then, estimates for subpopulations were summed (for instance, the “B cells” CIBERSORT estimate sums the estimates corresponding to naïve B cell, B memory cell, and plasma cell subpopulations).

### Comparison of MCP-counter TM sets with those from other methods

TM sets proposed by Bindea et al. (the “Immunome” TM set) and Yoshihara et al. (“ESTIMATE” TM set) were retrieved from the corresponding publication and the ESTIMATE R package, respectively. For the Immunome set, probe sets were used as TM for the Affymetrix Human Genome U133 Plus 2.0 and 133A microenvironment series and gene symbols for the HuGene 1.0 ST microenvironment series. For ESTIMATE, gene symbols were used for all three series. For each of the three microenvironment series, scores were computed (“Computation of MCP-counter scores” in the “Methods” section) from the TM sets proposed herein, the Immunome and ESTIMATE. For each TM set (columns) and each cell population represented in Fig. 4b (rows), the average score for the TM set in this cell population was computed. This vector of averaged score, called the “Pred” (for prediction) vector, was linearly mapped to a color code where the minimum value is blue, the maximum red, and the mean of the maximum and the minimum white. A “Truth” vector of colors, formed by the status of each cell population, was appended to each prediction vector (red, i.e., positive, if the cell population is supposed to express the genes in the TM set; blue, i.e., negative, if the cell population should not express genes in the TM set; and white if the cell population mixes both positive and negative cells). An accurate TM set should thus produce blue Truth is blue and red values when the Truth is red. White values of the Truth column are less informative as expression of the TM set would then depend both on the accuracy of the TM set and on the proportion of the corresponding cell population in the sample.

### Representative MCP-counter scores across cancers

In each of the three tumor series, samples were split according to their tumor type as represented in Fig. 4b. The median MCP-counter score was computed for each cell population in each tumor type, yielding three “median matrices” (one per technological platform). Since MCP-counter scores are expressed in arbitrary units which depend on the gene expression platform used, we Z-transformed each column of these three matrices (the mean of each column was subtracted on each platform and the resulting values were multiplied by the average of the three standard deviations observed across the three platforms for this column; Additional file 2: Figure S8). The resulting three matrices were averaged (omitting missing values in case of a cancer missing from one or two of the datasets; Fig. 5b).

### Prognostic value of MCP-counter scores

For a given cancer type, several datasets, sometimes based on different platforms, were collected. MCP-counter estimates were first computed for each dataset individually. The resulting scores were then Z-transformed for each dataset individually, leading to similar distributions of the scores across datasets. Then, univariate Cox proportional hazards models for OS were fitted separately in each dataset using the related Z-transformed MCP-counter scores. To aggregate the resulting estimates (beta values) across datasets, we used the meta-analytical R package meta (function metagen), using a fixed-effect model (as the scores in each series follow the same distribution). This function weights the independent estimates using an inverse-variance weighting.

### Microenvironment-based tumor classifications

MCP-counter estimates were first computed for each dataset individually. The resulting scores were then Z-transformed for each dataset individually, leading to similar distributions of the scores across datasets. Datasets from the same cancer were then merged and all MCP-counter variables were binarized using a median cut (leading to “high” and “low” samples for each variable and for each cancer according to their relative position from the cancer’s median value). We selected three tumor classifications from the literature (using B and T cells in lung adenocarcinoma, fibroblasts and cytotoxic lymphocytes in colorectal cancer, and macrophages and cytotoxic lymphocytes in breast cancer). For each of these three cancers, we concatenated the binarized scores for the two variables of interest, leading to four classes (high–high, high–low, low–high, low–low). The corresponding Kaplan–Meier curves for OS were then plotted and the *p* value of the corresponding log-rank test is reported.

## Additional files

Additional file 1: Table S1. Microenvironment cell populations (MCP) dataset. Discovery series. Affymetrix Human Genome U133 Plus 2.0. **Table S2.** The selected transcriptomic markers and their expression across MCP datasets. *fc* fold change, *auc* area under the ROC curve, *Fcspec* specific fold change (see “Methods”). *133P2* is the discovery MCP series; *133A* and *HG1* are the validation MCP series. *Restricted.TM.set* indicates whether the corresponding probe set is only in the complete set of markers (*No*) or if it is included in both the complete and the restricted set (*Yes*) (see Methods). **Table S3.** Result of the screening process on the discovery and validation series. For each non-root node (categories) represented in Additional file 2: Figure S1 or listed in the rows of Additional file 1: Table S13, and applying the screening process described in the “Results” and “Methods” sections, we report the number of markers identified on each series, the number of distinct datasets containing positive samples for this node, and the number of positive samples. The overlap between markers identified by this process on pairs or triplets of MCP series is reported with the corresponding Fisher’s exact test *p* value. **Table S4.** Microenvironment cell populations dataset. Validation series 1. Affymetrix 133A. **Table S5.** Microenvironment cell populations dataset. Validation series 2. Affymetrix HuGene ST 1.0. **Table S6.** Non-hematopoietic human tumor datasets. The numbers of GEP per expression platform and per tumor type are listed, as well as the number samples annotated for overall survival (OS). **Table S7.** Non-hematopoietic human primary tumors dataset 1. Affymetrix Human Genome U133 Plus 2.0. **Table S8.** Non-hematopoietic human primary tumors dataset 2. Affymetrix 133A**. Table S9** Non-hematopoietic human primary tumors dataset 3. TCGA, Illumina HiSeq**. Table S10** mRNA from purified cell populations: mixture proportions (microarray dataset). **Table S11.** mRNA from purified cell populations: mixture proportions (qPCR dataset). **Table S12.** Non-hematopoietic human primary tumors meta-dataset 4. Other gene expression platforms, samples with OS annotations. **Table S13.** Relationship between MCP sample labels and categories represented in Additional file 2: Figure S11. MCP sample labels are in columns and categories in lines. A “1” designates positive labels and indicates inclusion of the label in the category (which means that all cell populations contained in the sample are in the category); “0” designates negative labels and indicates strict separation of the label from the category (which means that no cell type from the sample is in the category); −1 designates excluded labels (for “mixed samples”) and indicates contamination of the sample by the category (which means that some cells composing the sample are in the category and some are outside the category). **Table S14.** Author-reported tissue types, recoded tissue types, and anatomical system of non-diseased human tissue samples (dataset GSE7307). **Table S15.** List of TaqMan probes used in qPCR analyses. **Table S16.** Antibodies and experimental conditions used for immunohistochemical analyses. (XLSX 1568 kb)
Additional file 2: Figure S1. Hierachization of samples’ recoded phenotypes. The sample labels present in the discovery or validation series are all represented on the graph, as well as manually encoded higher-level categories. *Arrows* denote the relationship “includes”. A second type of relationship, “mixed samples” (see “Methods” for its use and definition) is not represented here but is detailed in Additional file 1: Table S13. Cancer cell lines from various organs were aggregated under a single label in this figure for simplicity. **Figure S2.** Consistency between MCP sample hierarchization and unsupervised data representation. Principal component analyses of MCP samples across the three MCP datasets based on the features whose standard deviation ranked among the top 5 %. Samples are colored according to their phenotype. **Figure S3.** Reproducibility of transcriptomic marker expression patterns between the discovery and validation series. Transcriptomic marker expression quartiles in peripheral blood mononuclear cells (PBMC), CD3^+^ T cells, CD4^+^ T cells, CD8^+^ T cells, NK cells, Tγδ cells, B cells, pDC (plasmacytoid Dendritic Cells), Granulocytes, Neutrophils, Eosinophils, Monocytes, Macrophages, myeloid dendritic cells (mDC), mast cells, endothelial cells, fibroblasts, and cancer cell lines in the MCP discovery series and MCP validation series. **Figure S4.** AUC for each MCP-counter score on MCP discovery and validation series. The TM sets used here are the “complete” sets, which do not use any information from the validation series. **Figure S5.** Expression pattern of the BLK gene across MCP samples. **Figure S6.** Coexpression of transcriptomic markers specific for the same cell population in the three MCP datasets. Heat map representation of correlation matrices. The TM sets used here are the “complete” sets, which do not use any information from the validation series. **Figure S7.** Coexpression of transcriptomic markers specific for the same cell population in the three tumor datasets. Heat map representation of TM correlation matrices. TM were ordered using hierarchical clustering with complete linkage and Pearson’s “distance”. **Figure S8.** Reproducibility of the coexpression patterns within malignancies. Heat map representation of TM correlation matrices within each malignancy. The color scale is the same as in Additional file 2: Figure S6 as well as the order of the features. **Figure S9.** Distribution of summarized TM sets on microenvironment series. Boxplots showing the distribution of scores computed with TM sets from Bindea et al. (*left*) and MCP-counter complete TM sets (*right*) across the three MCP series. For each TM set, boxplots are colored *red* for “positive” samples, *blue* for “negative” samples, and gray for “mixed” samples. **Figure S10.** Reproducibility of the malignancy mean abundance estimates across the three primary human tumor meta-datasets. Means of MCP-counter scores across malignant tissues. For each platform, columns were Z-transformed. *Dark red* denotes highest and *dark blue* lowest Z-scores. A *gray line* indicates that the corresponding malignancy is not represented in the meta dataset. (PPTX 9607 kb)

## Abbreviations

AUC: Area under the ROC curve
FC: Fold change
fRMA: Frozen robust multiarray average
GEO: Gene Expression Omnibus
HUVEC: Human umbilical vein endothelial cell
IHC: Immunohistochemistry
MCP: Microenvironment Cell Populations
NK: Natural killer
OS: Overall survival
PBMC: Peripheral blood mononuclear cell
PMN: Polymorphonuclear cell
qPCR: Quantitative polymerase chain reaction
sFC: Specific fold change
TCGA: The Cancer Genome Atlas
TM: Transcriptomic marker
