# Erratum to: Estimating the population abundance of tissue-infiltrating immune and stromal cell populations using gene expression

**DOI:** 10.1186/s13059-016-1113-y

**Published:** 2016-12-01

**Authors:** Etienne Becht, Nicolas A. Giraldo, Laetitia Lacroix, Bénédicte Buttard, Nabila Elarouci, Florent Petitprez, Janick Selves, Pierre Laurent-Puig, Catherine Sautès-Fridman, Wolf H. Fridman, Aurélien de Reyniès

**Affiliations:** 1 INSERM UMR_S 1138, Cancer, Immune Control and Escape, Cordeliers Research Centre, Paris, France; 2 Université Paris Descartes, Paris, France; 3 Université Pierre et Marie Curie, Paris, France; 4 Programme Cartes d’Identité des Tumeurs, Ligue Nationale Contre le Cancer, Paris, France; 5 Centre de Recherche en Cancérologie de Toulouse, Unité Mixte de Recherche, 1037 INSERM, Université Toulouse III, Toulouse, France; 6 Department of Pathology, Centre Hospitalier Universitaire de Toulouse, Toulouse, France; 7 INSERM, UMR_S1147, Paris, France

## Erratum

After the publication of this work [1] it was noticed that the legend of Fig. 1a was incorrect. The correct legend should read: Flowchart of MCP-counter’s development and validation. It was also noted that in Fig. 2c the name of the last two genes was partially removed. The horizontal lines that were delineating the specificity of the displayed genes are missing. The corrected Fig. 2c is noted below.

**Fig. 2:**
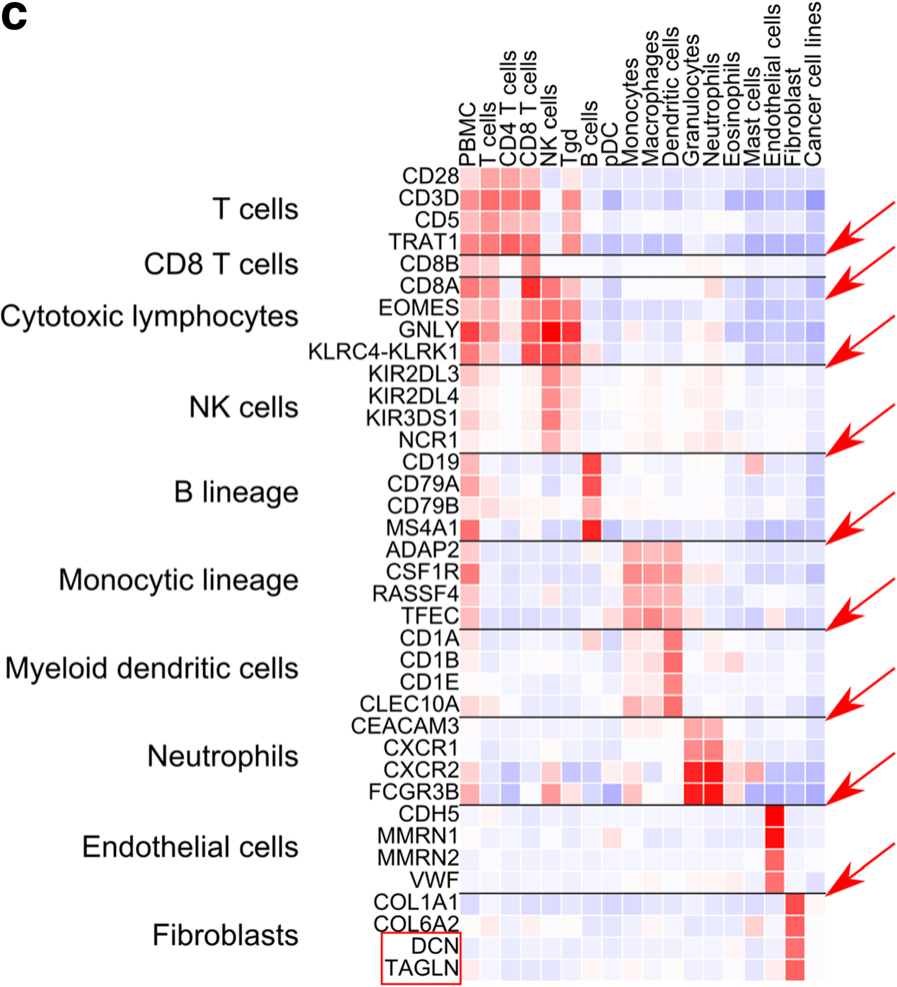
Identification and qualitative validation of transcriptomic markers. **a** The MCP discovery series. *pDC* plasmacytoid dendritic cell, *PBMC* peripheral blood mononuclear cell. **b** Quartiles of MCP-counter scores on positive and control samples in the discovery and validation microenvironment series. *Gray* indicates missing values. **c** Representative transcriptomic markers and their corresponding expression patterns in the MCP discovery series

The equal contributors symbol was missing for authors Aurélien de Reyniès and Wolf H. Fridman. This has now been corrected.

In the PDF version of the article, equation 5 in the section ‘Correlation profiles of TM in microenvironment and tumor datasets’ is incorrect. Please see the correct equation below:

5 $$ {\uppi}_{\mathrm{k},\mathrm{j}}=\frac{{\mathrm{f}}_{\mathrm{S},\mathrm{j}}}{{\mathrm{f}}_k}=\frac{{\mathrm{g}}_{S,j}}{{\mathrm{g}}_k} $$

The publisher apologises for these errors.
