# Supplementary material for: Estimating the population abundance of tissue-infiltrating immune and stromal cell populations using gene expression
Source: Genome Biol. 2016 Oct 20;17:218. doi: 10.1186/s13059-016-1070-5 (PMC5073889; doi:10.1186/s13059-016-1070-5)
Supplement: Additional file 2: Figure S1. — Hierachization of samples’ recoded phenotypes. The sample labels present in the discovery or validation series are all represented on the graph, as well as manually encoded higher-level categories. Arrows denote the relationship “includes”. A second type of relationship, “mixed samples” (see “Methods” for its use and definition) is not represented here but is detailed in Additional file 1: Table S13. Cancer cell lines from various organs were aggregated under a single label in this figure for simplicity. Figure S2. Consistency between MCP sample hierarchization and unsupervised data representation. Principal component analyses of MCP samples across the three MCP datasets based on the features whose standard deviation ranked among the top 5 %. Samples are colored according to their phenotype. Figure S3. Reproducibility of transcriptomic marker expression patterns between the discovery and validation series. Transcriptomic marker expression quartiles in peripheral blood mononuclear cells (PBMC), CD3+ T cells, CD4+ T cells, CD8+ T cells, NK cells, Tγδ cells, B cells, pDC (plasmacytoid Dendritic Cells), Granulocytes, Neutrophils, Eosinophils, Monocytes, Macrophages, myeloid dendritic cells (mDC), mast cells, endothelial cells, fibroblasts, and cancer cell lines in the MCP discovery series and MCP validation series. Figure S4. AUC for each MCP-counter score on MCP discovery and validation series. The TM sets used here are the “complete” sets, which do not use any information from the validation series. Figure S5. Expression pattern of the BLK gene across MCP samples. Figure S6. Coexpression of transcriptomic markers specific for the same cell population in the three MCP datasets. Heat map representation of correlation matrices. The TM sets used here are the “complete” sets, which do not use any information from the validation series. Figure S7. Coexpression of transcriptomic markers specific for the same cell population in the three tumor datasets. Heat map representatio [file 13059_2016_1070_MOESM2_ESM.pptx]

## Slide 1
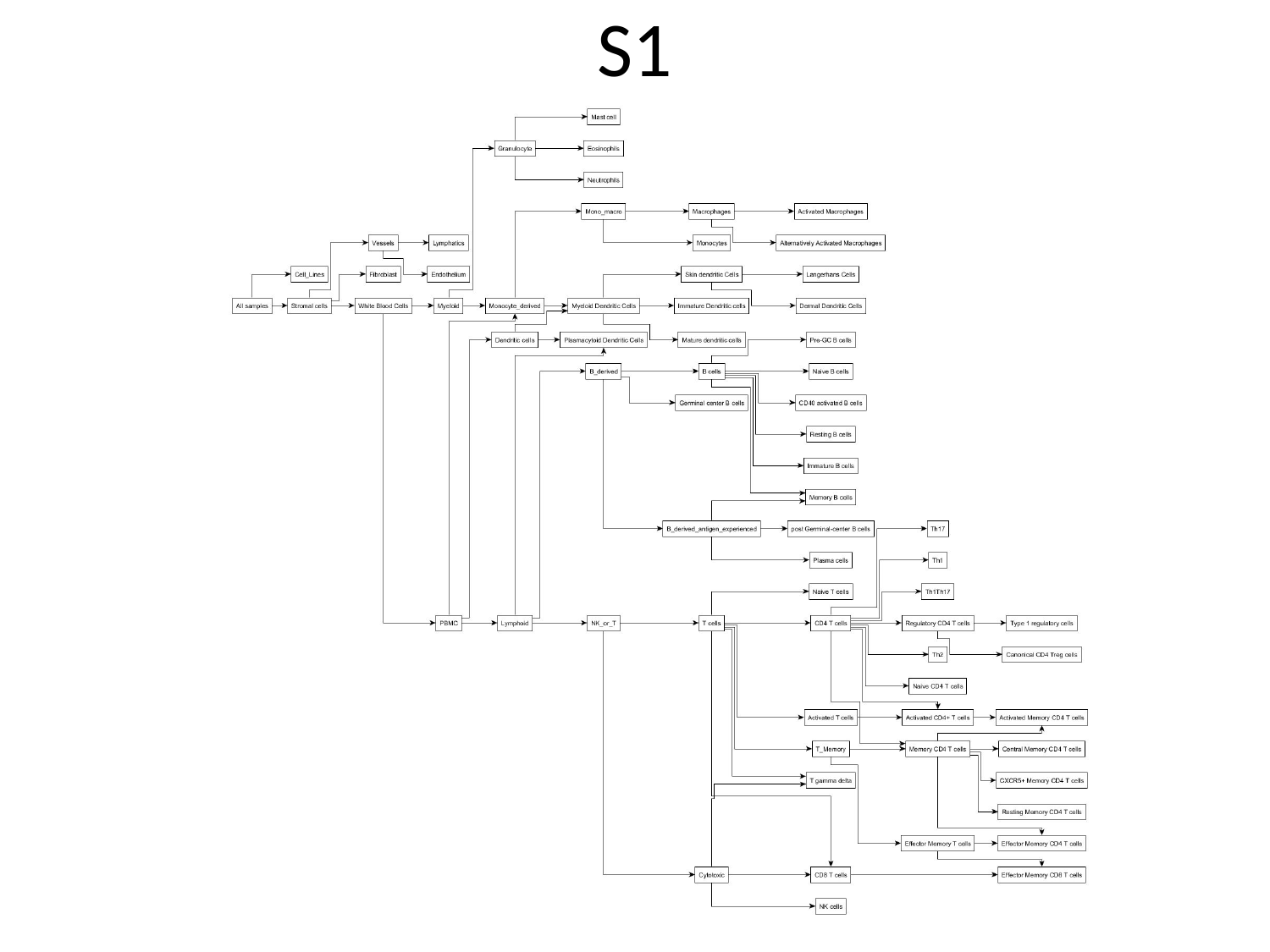

# S1

## Slide 2
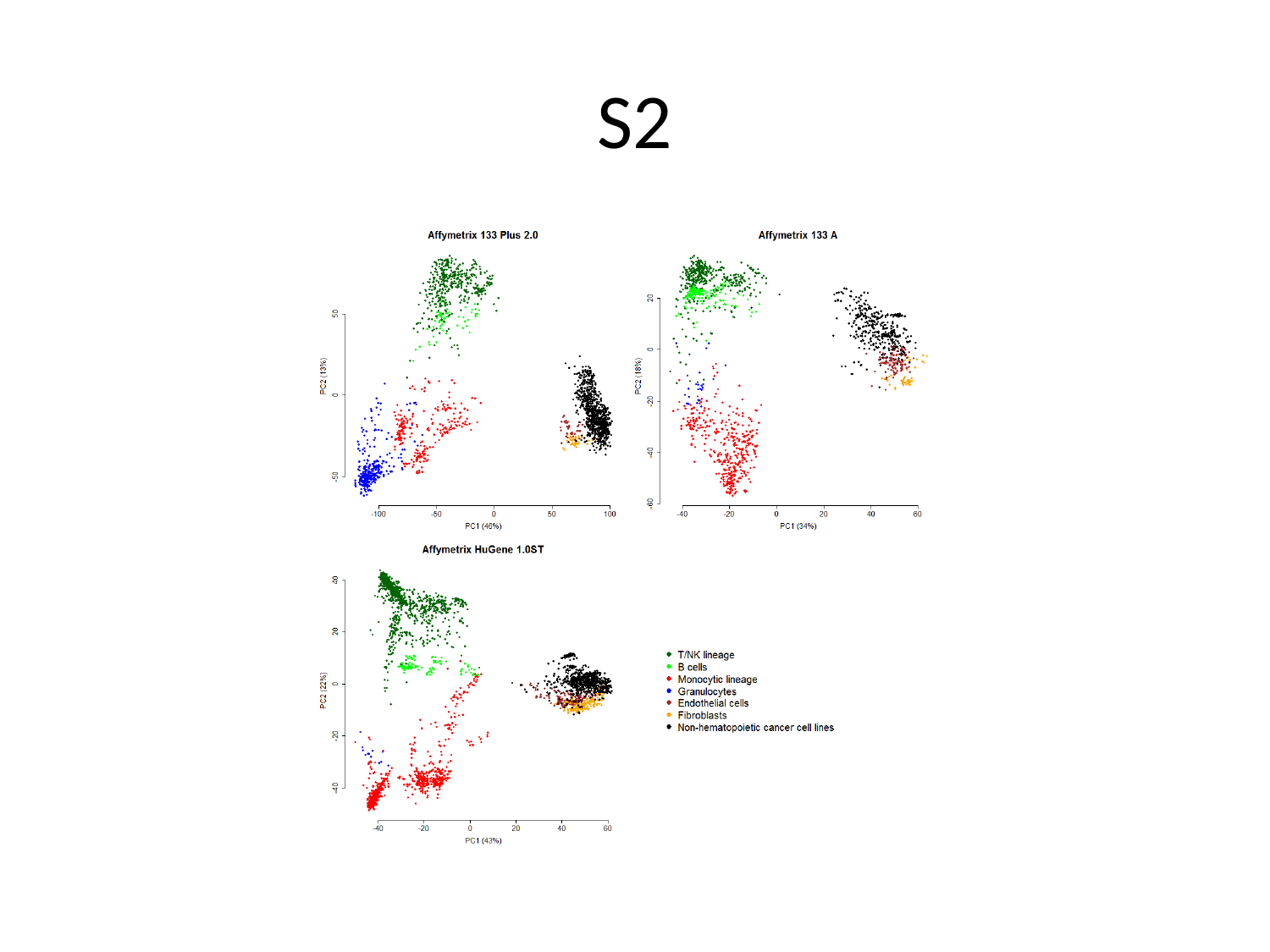

# S2

## Slide 3
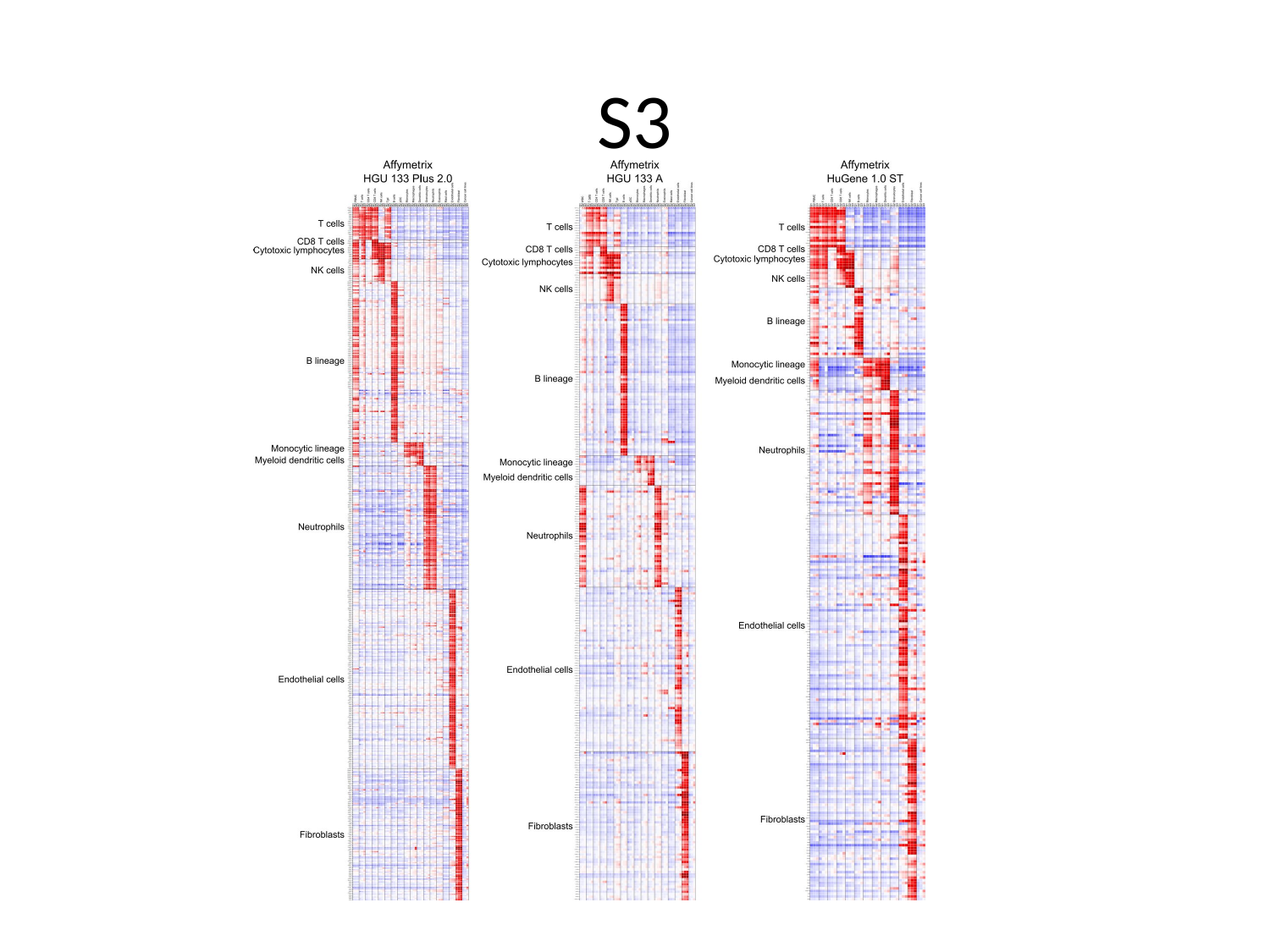

# S3

## Slide 4
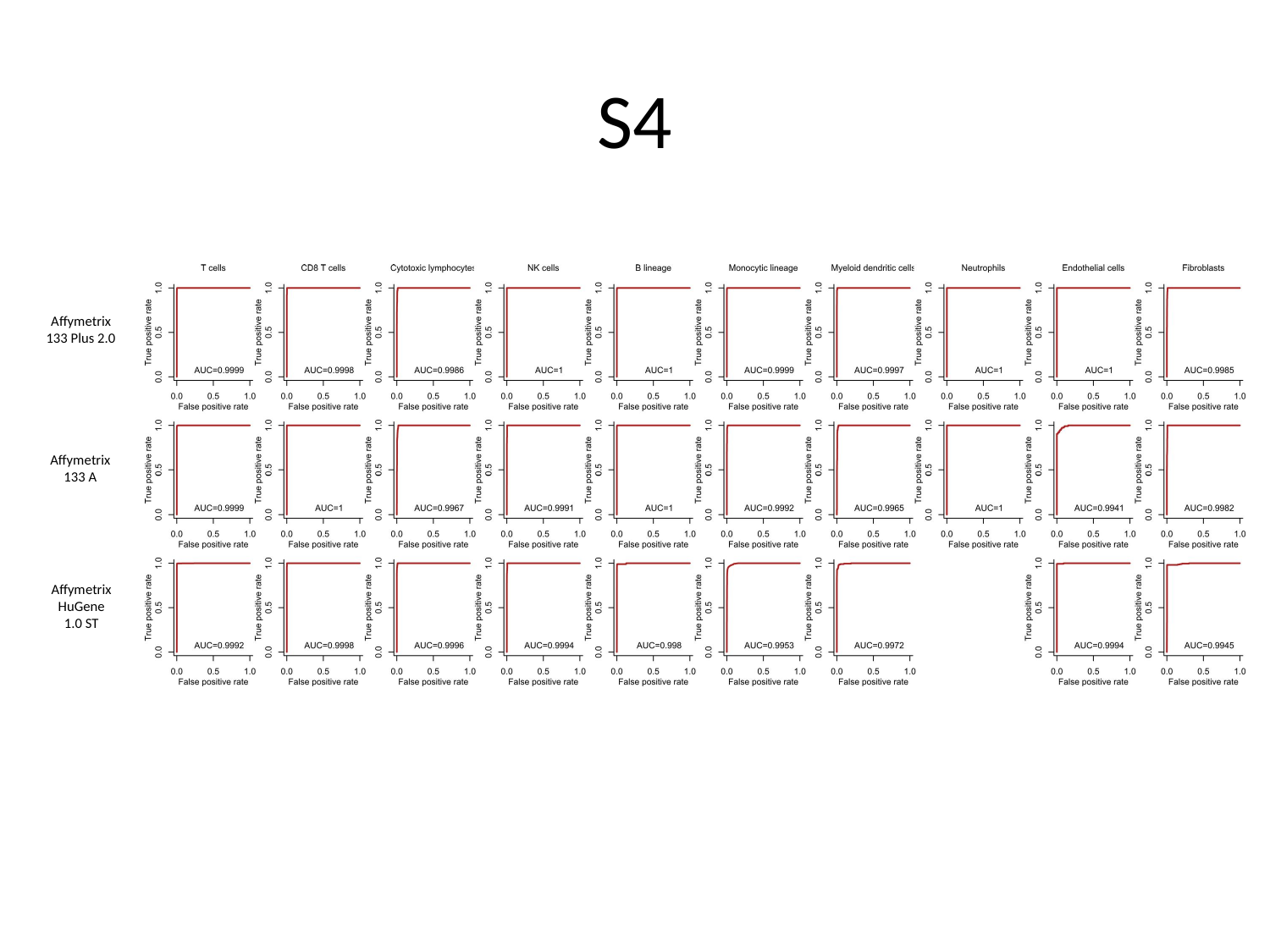

# S4
Affymetrix
133 Plus 2.0
Affymetrix
133 A
Affymetrix
HuGene
1.0 ST

## Slide 5
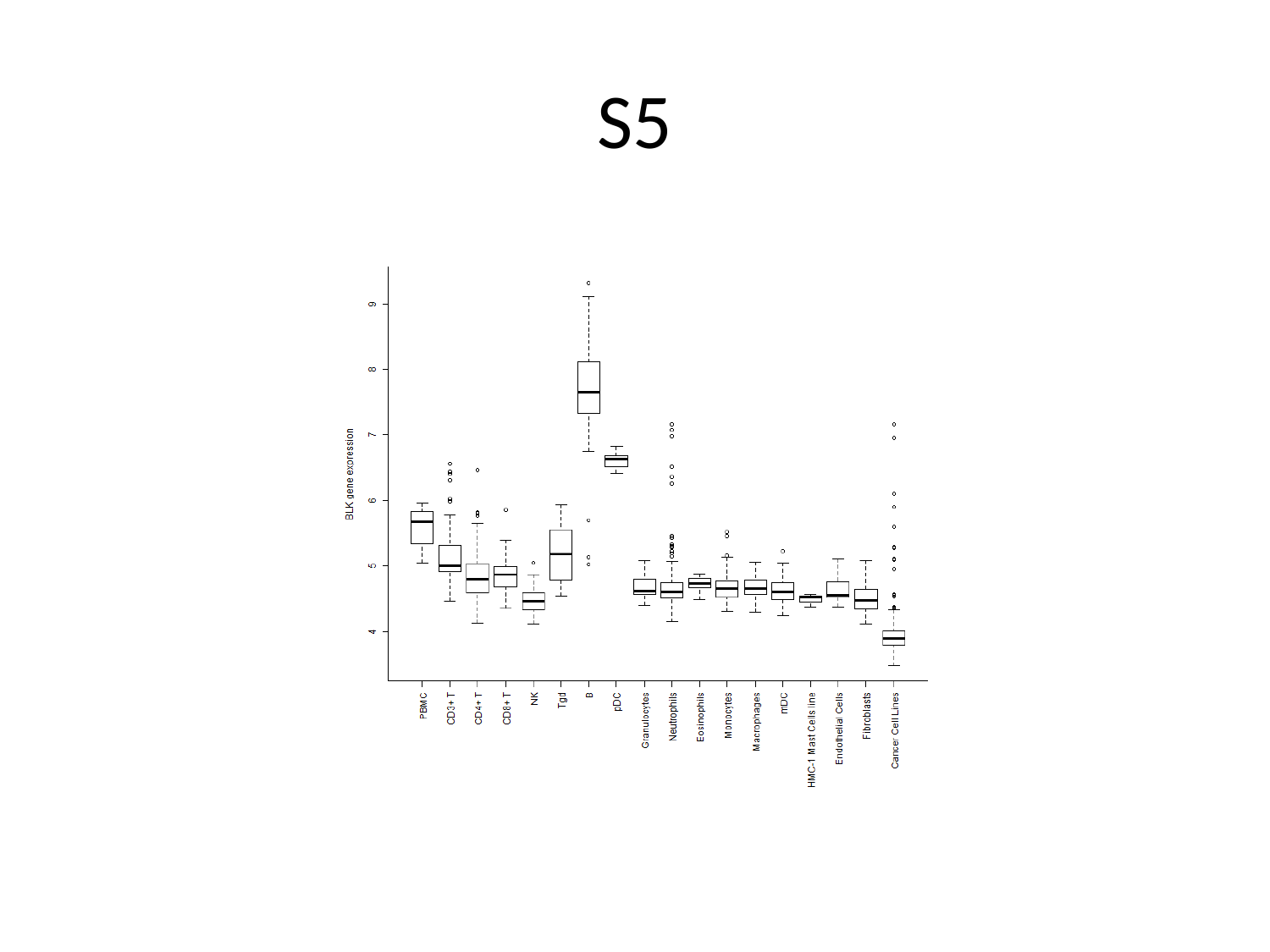

# S5

## Slide 6
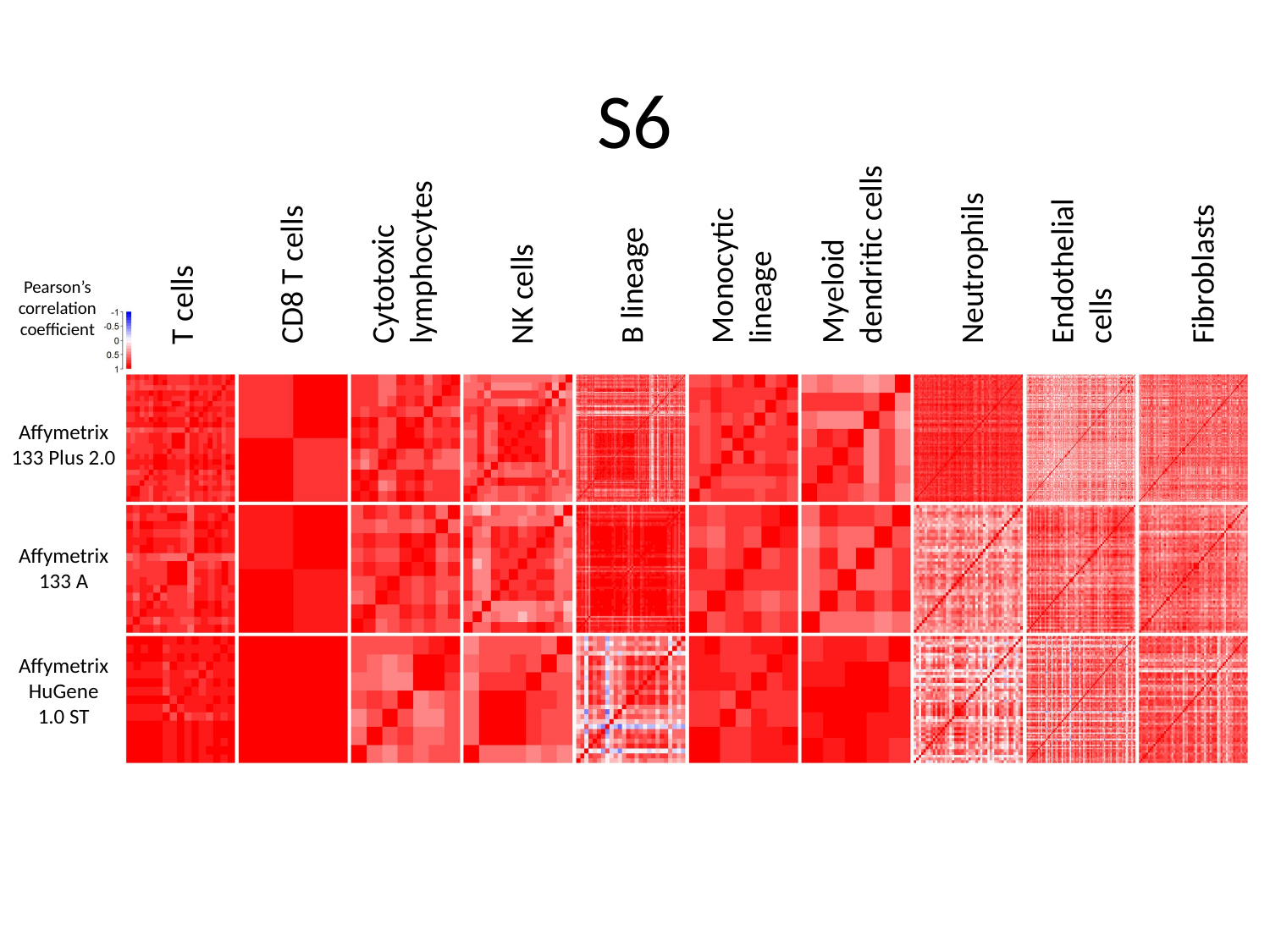

# S6
Myeloid
dendritic cells
Cytotoxic
lymphocytes
Endothelial
cells
Monocytic
lineage
Neutrophils
Fibroblasts
CD8 T cells
B lineage
NK cells
Pearson’s
correlation
coefficient
T cells
Affymetrix
133 Plus 2.0
Affymetrix
133 A
Affymetrix
HuGene
1.0 ST

## Slide 7
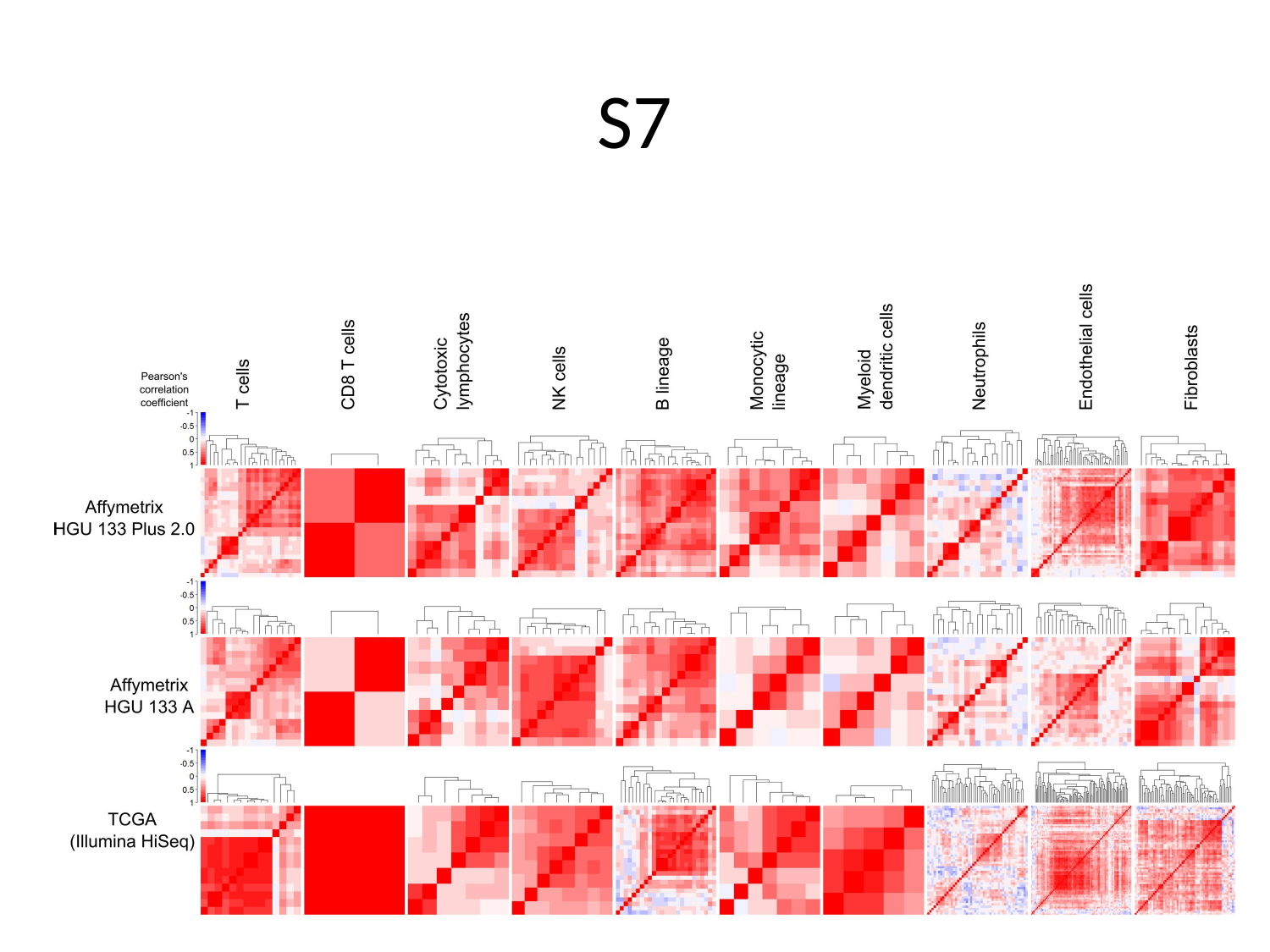

# S7

## Slide 8
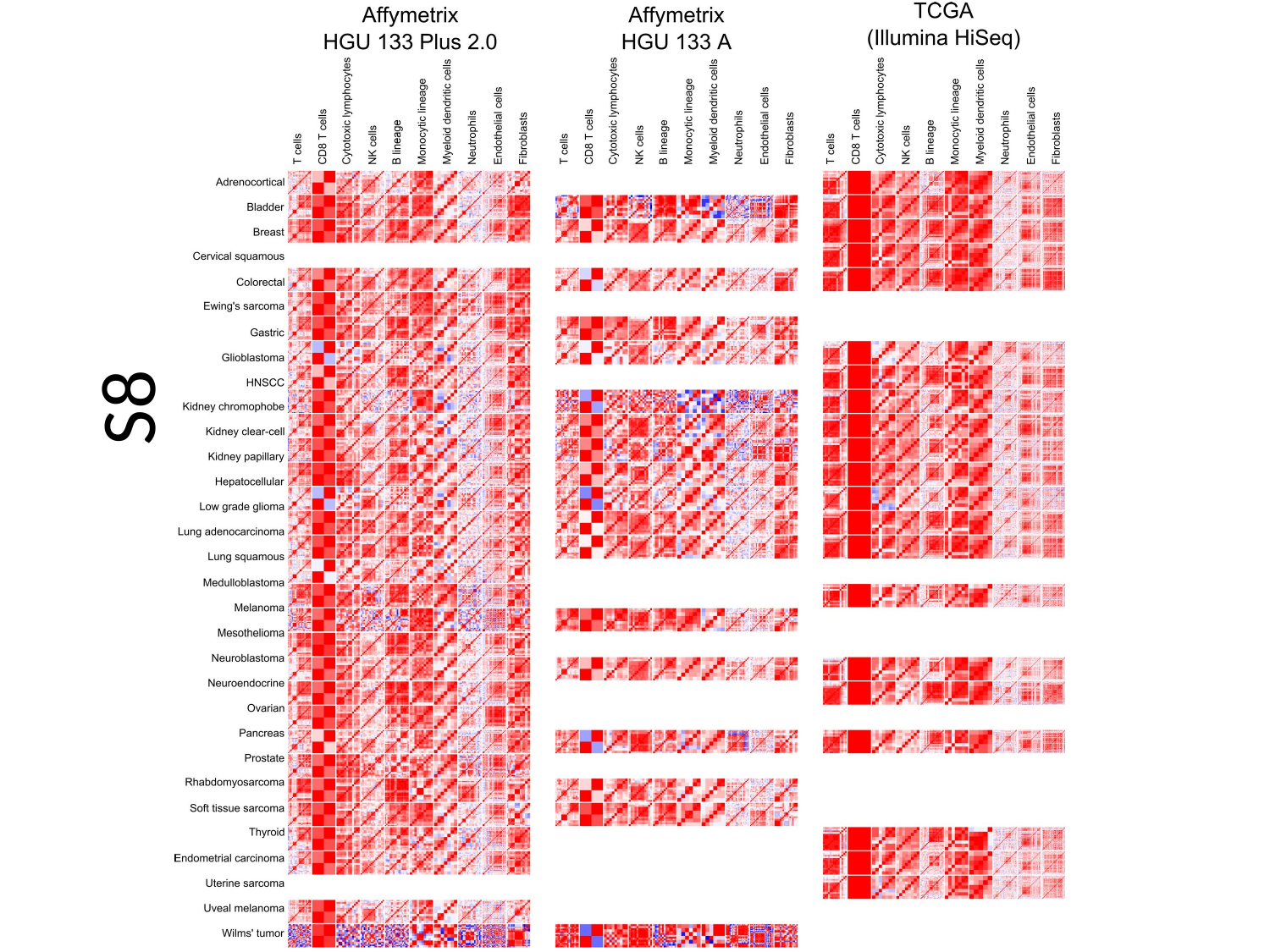

# S8

## Slide 9
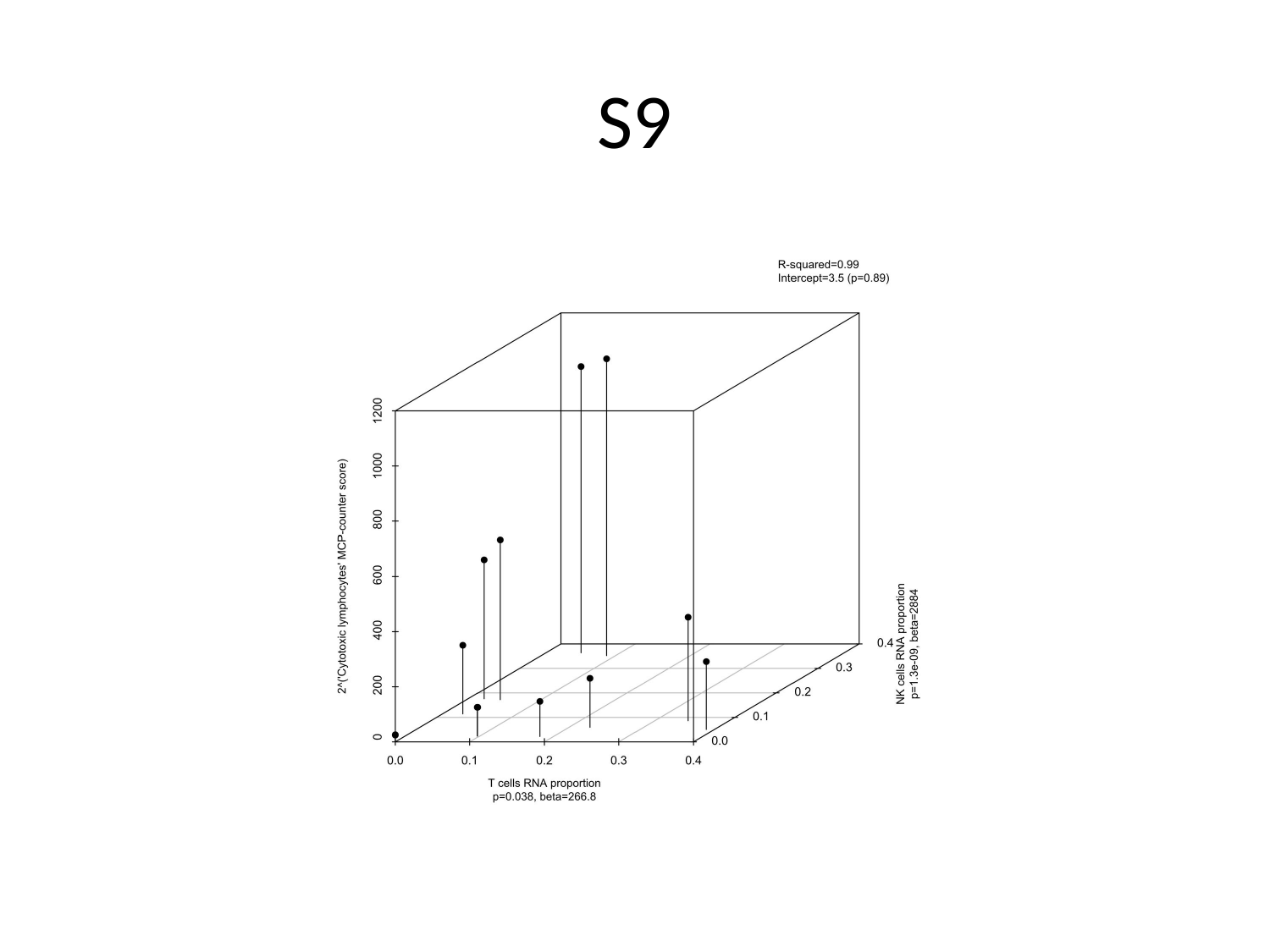

# S9

## Slide 10
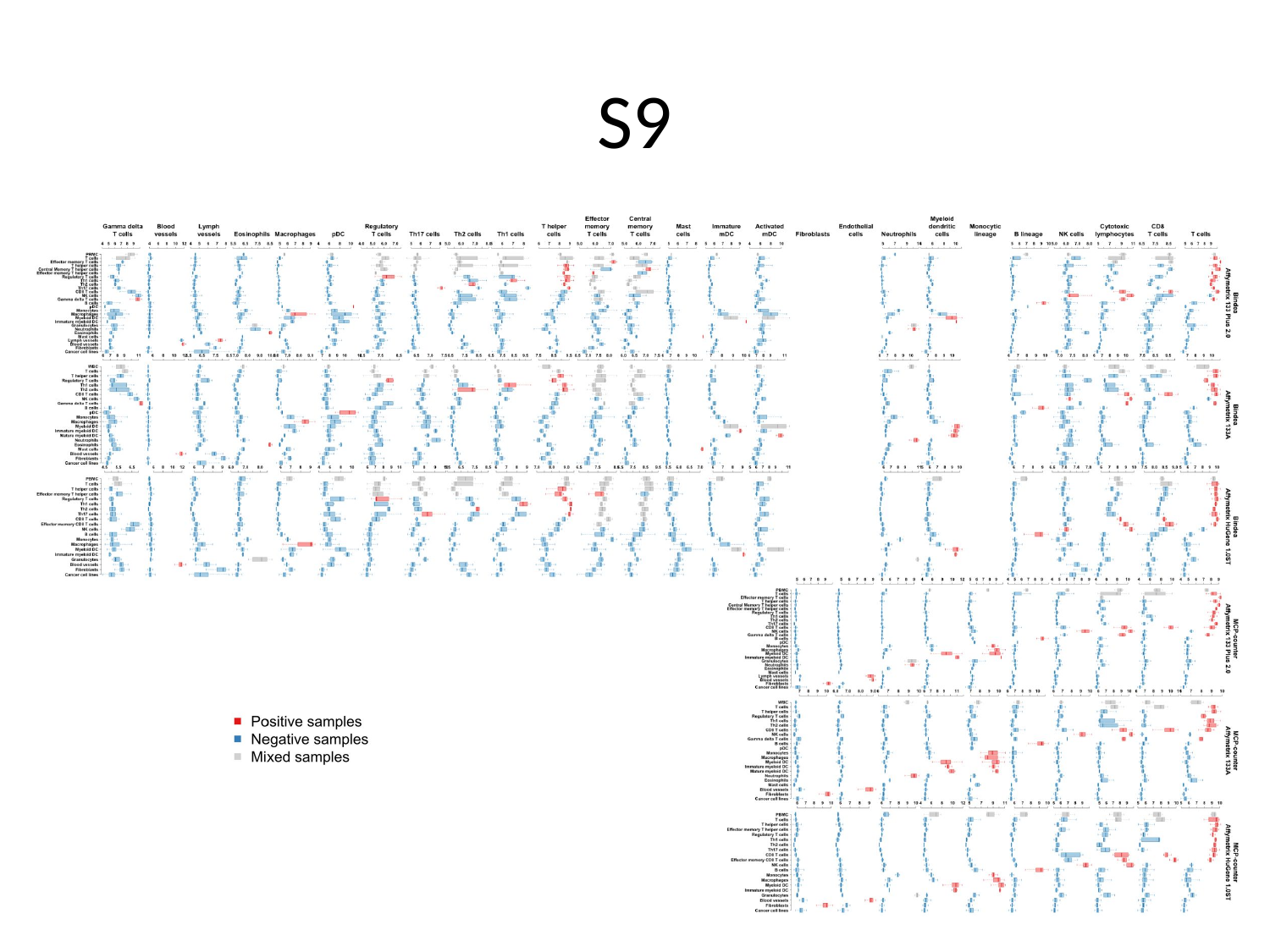

# S9

## Slide 11
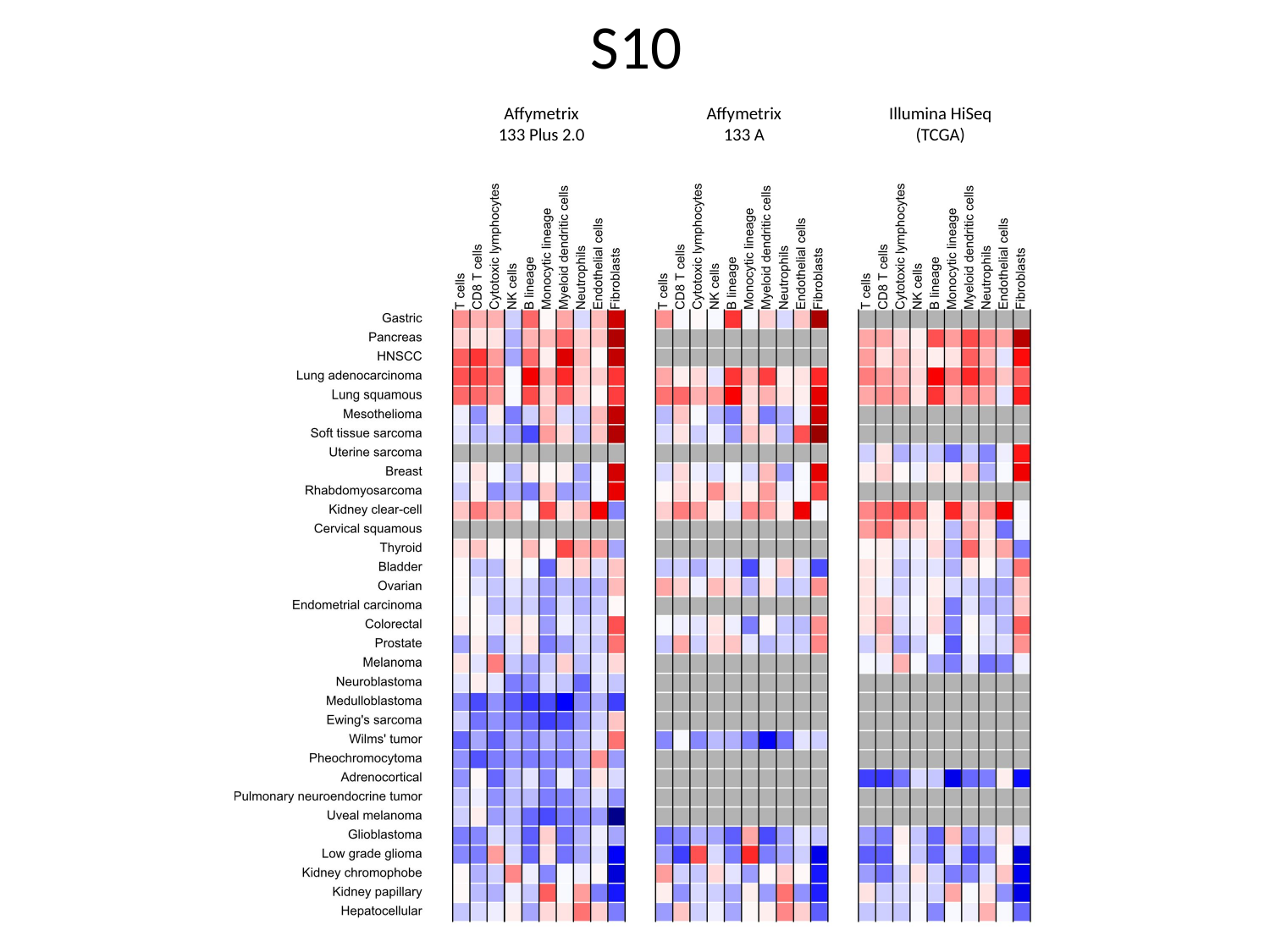

# S10
Affymetrix
133 Plus 2.0
Affymetrix
133 A
Illumina HiSeq
(TCGA)
